# A ZNF-nanobody fusion reveals SUMOylation-dependent changes in p53 protein localization

**DOI:** 10.1016/j.isci.2026.115673

**Published:** 2026-04-09

**Authors:** Antoine Y. Bouchard, Valérie C. Cabana, Julien Plamondon, Chongyang Li, Anaïs J.I. Vivet, Sylvie Mader, Pierre Thibault, Marc P. Lussier, Laurent Cappadocia

**Affiliations:** 1 Département de Chimie, Université du Québec à Montréal, 2101 Jeanne-Mance Street, Montréal, Québec H2X 2J6, Canada; 2 Centre d’Excellence en Recherche sur les Maladies Orphelines – Fondation Courtois (CERMO-FC), Faculté des Sciences, Université du Québec à Montréal, 2101 Jeanne-Mance Street, Montréal, Québec H2X 2J6, Canada; 3 Regroupement québécois de recherche sur la fonction, l’ingénierie et les applications des protéines (PROTEO), 2101 Jeanne-Mance Street, Montréal, Québec H2X 2J6, Canada; 4 Institut de Recherche en Immunologie et Cancérologie (IRIC), Université de Montréal, 2950 Chem. de Polytechnique, Montréal, Québec H3T 1J4, Canada; 5 Département de Biochimie et Biologie Moléculaire, Université de Montréal, 5155 Chemin de la Rampe, Montréal, Québec H3T 1J4, Canada; 6 Département de Chimie, Université de Montréal, 1375 Avenue Thérèse-Lavoie-Roux, Montréal, Québec H2V 0B3, Canada

**Keywords:** biological sciences

## Abstract

SUMOylation is a post-translational modification regulating protein localization, stability, and activity, with effects varying depending on the conjugated SUMO protein and type of SUMOylation. To determine how enhanced SUMOylation affects protein localization, we fused ZNF, a SUMOylation tag derived from SUMO E3 ligase ZNF451 that biases substrates toward SUMO2/3, to the GFP-binding nanobody vhhGFP4 (VHH), creating VHH-ZNF to drive SUMOylation of GFP-tagged substrates in *trans*. *In vitro*, VHH-ZNF increased SUMO2/3 modification of p53-GFP, preferentially generating polySUMO2 chains at the canonical K386 site. In HEK293 cells co-expressing p53-GFP and VHH-ZNF, immunoblotting and proteomics confirmed increased SUMO2/3 conjugation of p53 at K386. Fluorescence microscopy analyses revealed that SUMOylated p53 transitions from a diffuse nuclear distribution to SUMO-positive nuclear foci that partially overlap with promyelocytic leukemia (PML) and, less so, to 53BP1 nuclear bodies. Overall, we developed a method to increase the SUMOylation of GFP-tagged proteins and to visualize SUMOylation-dependent relocalization in cells.

## Highlights

• Elaboration of a SUMOylation tool composed of a ZNF tag fused to a VHH nanobody
• VHH-ZNF increases SUMOylation of GFP-tagged substrate *in vitro* and in HEK293 cells
• VHH-ZNF increases poly-SUMOylation of p53 at the consensus SUMOylation site K386
• SUMOylation of p53 promotes partial localization to PML nuclear bodies

## Graphical abstract

**Figure:**
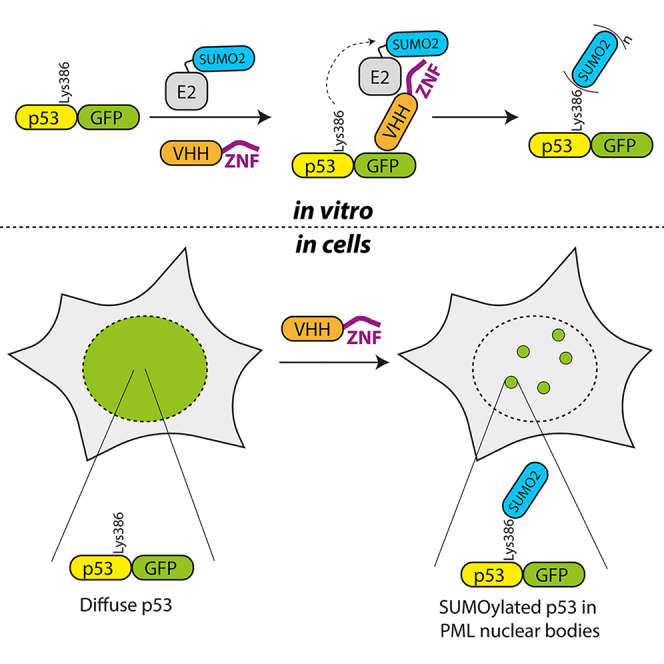

## Introduction

SUMOylation is a reversible post-translational modification that shapes the abundance, activity, and localization of numerous nuclear proteins, influencing processes such as gene transcription,^1^ DNA repair,^2^ and stress response.^3^ SUMOylation is also an ATP-dependent reaction involving the sequential action of three enzymes.^4^ First, the SUMO E1 activating enzyme (SAE1/SAE2) activates SUMO and forms a thioester bond with an exposed diglycine motif at its C-terminus.^5^^,^^6^ SUMO is then transferred to a SUMO E2 conjugating enzyme (Ubc9),^7^ before being conjugated to a lysine residue of a substrate typically located within a SUMOylation consensus motif ψ-K-x-E/D, where ψ is a hydrophobic amino acid, K is the target lysine, x is any amino acid, and E/D are glutamate and aspartate residues.^8^ SUMO E3 ligases accelerate the SUMOylation reaction by binding both the substrate and the SUMO-charged E2 (E2∼SUMO) and facilitating the transfer of SUMO to the substrate.^9^^,^^10^ While only a single SUMO E1 and SUMO E2 exist in humans, several SUMO E3 ligases have been discovered,^9^ each with their own substrate pool.^11^^,^^12^ The SUMOylation reaction can be reversed by the action of SUMO-specific proteases (SENPs), which remove SUMOs from a substrate to regenerate SUMO and the unconjugated substrate.^13^

The human genome encodes three conjugation-competent SUMO paralogs: SUMO1, SUMO2, and SUMO3.^14^ SUMO2 and SUMO3 are 97% identical and frequently considered together as SUMO2/3.^15^ Unlike SUMO1, SUMO2/3 contain an internal consensus site that enables chain formation on substrates.^16^ Functionally, mono- and multi-SUMOylation (the modification of one or multiple lysine residues), often rewire protein-protein interactions,^17^^,^^18^^,^^19^ whereas polySUMOylation (the addition of multiple SUMOs on a single lysine to form a chain) can recruit SUMO-targeted ubiquitin ligases (STUbLs), leading to ubiquitination and, in some contexts, proteasomal degradation.^20^

We recently developed a small and lysine-less ZNF SUMOylation tag (residues 25–56 of ZNF451) that retains E3 activity and selectively enhances SUMO2/3 conjugation of fused substrates in *cis*.^21^^,^^22^ When appended to p53, this tag increased SUMOylation and attenuated p53-dependent transcription in luciferase assays, suggesting that altered modification status, potentially via changes in subcellular localization, underlies the functional effect. SUMOylation is indeed well known to influence localization: for example, it drives nuclear export of CRM1 and Smad4 in specific contexts,^23^^,^^24^ and targets proteins such as DAXX and SP100 to promyelocytic leukemia nuclear bodies (PML NBs).^25^^,^^26^^,^^27^ Constituting hubs for many SUMOylated proteins, PML-NBs are membrane-less nuclear organelles that play roles ranging from DNA damage response to transcriptional regulation and stress response.^28^^,^^29^ Regarding p53 localization, reports are mixed; SUMOylation has been linked to both nuclear export^30^ and recruitment to PML NBs via SUMO-dependent,^31^ or SUMO-independent mechanisms.^32^

To investigate the changes in localization associated with ZNF-induced SUMOylation, we fused the ZNF tag to a vhhGFP4 nanobody (VHH),^33^ creating VHH-ZNF to SUMOylate GFP-tagged substrates in *trans* and with tunable stoichiometry. Through *in vitro* assays, we show that VHH-ZNF promotes the SUMOylation of GFP-fused p53 with a clear preference for SUMO2 instead of SUMO1. Although VHH-ZNF is unable to promote the SUMOylation of HA-p53, the addition of p53-GFP to the reaction increases its SUMOylation, suggesting that VHH-ZNF can target other subunits of mixed p53 complexes in a proximity-dependent manner *in vitro*. We confirmed, using immunoprecipitation and immunoblotting, that VHH-ZNF is also effective at promoting the SUMOylation of p53-GFP in HEK293 cells. Mass spectrometry experiments further reveal that lysine 386 constitutes the main acceptor site of SUMOylation of p53 both *in vitro* and in cells. Finally, we show that SUMOylated p53 primarily appears in nuclear foci overlapping with SUMO, unlike the unmodified protein, which is mostly diffused in the nucleus. Notably, VHH-ZNF greatly increase the overlap of PML NBs with p53-GFP, suggesting that SUMOylation of p53 promotes its recruitment to PML NBs, but also, in smaller proportions, to other NBs such as 53BP1 NBs. Together, these findings establish VHH-ZNF as a useful tool to boost SUMOylation of GFP-tagged proteins and to interrogate SUMOylation-dependent changes in subcellular localization.

## Results

### A VHH-ZNF fusion increases the SUMOylation of GFP-tagged p53

**Figure 1:**
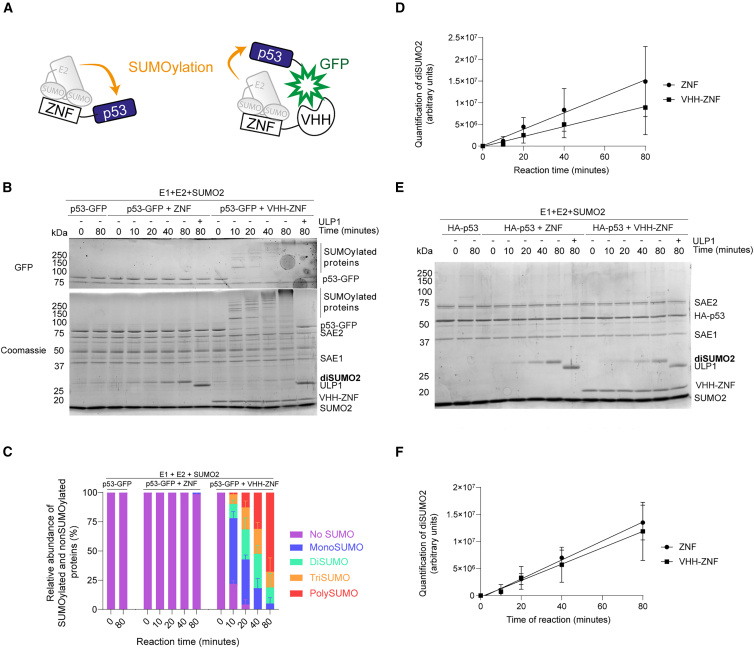
VHH-ZNF specifically increases SUMOylation of GFP-fused p53 (A) Pictograms showing the SUMOylation of p53 in *cis* in the context of a ZNF-p53 fusion (left) and the SUMOylation in *trans* of p53-GFP by the VHH-ZNF nanobody (right). (B and E) *In vitro* assays comparing the SUMOylation of (B) p53-GFP with ZNF or VHH-ZNF and (E) HA-p53 with ZNF or VHH-ZNF. Both assays were performed using 100 nM E1 (SAE1/SAE2), 100 nM E2 (Ubc9, not visible on the gels), and 50 μM SUMO2. Reactions were started with 2 mM ATP and stopped at each time point using Laemmli buffer. After 80 min, 1 μM ULP1 was added for 10 min, after which the reaction was stopped with Laemmli buffer. Reactions were loaded on a 12% SDS-PAGE. GFP signal was imaged using blue light illumination on a ChemiDoc. Gels were then stained with Coomassie blue. Gels are a representative of three independent experiments (*N* = 3). (C) Relative intensity of the SUMOylated and non-SUMOylated p53-GFP at each time point obtained from the quantification of the gel presented in (B). (D and F) Quantification of the diSUMO2 band intensity for the reactions involving ZNF or VHH-ZNF with (D) p53-GFP obtained from (B), or (F) HA-p53 obtained from (E). All results are mean ± SD from *N* = 3.

Intrigued by the capacity of the ZNF tag to regulate p53 activity without substantially changing its abundance,^21^ we wondered whether this effect could be due to changes in p53 localization as suggested by several reports.^30^^,^^31^^,^^32^ We thus devised a strategy to promote the SUMOylation of a p53-GFP construct *in trans* using a fusion between the ZNF tag and vhhGFP4, a VHH nanobody that specifically recognizes GFP^33^ (Figure 1A).

We started by comparing the SUMOylation of p53-GFP *in vitro* in the presence of ZNF or a ZNF-tagged vhhGFP4 nanobody, hereafter termed VHH-ZNF. Following the reaction, we used blue epi illumination on the SDS-polyacrylamide gel to excite and detect the GFP-tagged protein (Figure 1B, top) and thus selectively detect p53-GFP. We then stained the gel with Coomassie blue to visualize all proteins in the assay (Figure 1B, bottom). After 80 min, in the presence of VHH-ZNF, the unmodified p53-GFP protein band completely disappeared. In the absence of VHH-ZNF, however, the unmodified p53-GFP band intensity did not decrease, even after 80 min. Protein bands of high molecular weight appeared in a time-dependent manner for the p53-GFP plus VHH-ZNF condition only, similar to our previous observations with ZNF-p53^21^ (Figure 1B). Treating those reactions with the SUMO protease ULP1 regenerated the unmodified p53-GFP band, confirming that the high molecular weight bands genuinely correspond to SUMOylated p53-GFP (Figure 1B). Taken together, these results suggest that VHH-ZNF promotes p53-GFP SUMOylation *in trans*.

Next, we quantified the relative band intensity at each time point, distinguishing unmodified p53-GFP (no SUMO), monoSUMOylated, diSUMOylated, triSUMOylated, and higher order multi/polySUMOylated forms. Quantification confirmed that p53-GFP rapidly undergoes rapid multi/polySUMOylation in the presence of VHH-GFP (Figure 1C). In similar conditions, ZNF was unable to promote even strong monoSUMOylation of p53-GFP, confirming the capacity of VHH-ZNF to efficiently promote SUMOylation of a GFP-fused substrate. Lastly, quantification of the accumulation of diSUMO2 at the bottom of the gel for p53-GFP with ZNF or VHH-ZNF (Figure 1B, bottom) revealed no major difference between the two conditions (Figure 1D). Overall, these results suggest that VHH-ZNF can promote the efficient SUMOylation of a GFP-tagged protein *in vitro*.

To ensure that VHH-ZNF is specifically targeting substrates in a GFP-dependent manner, we assessed the SUMOylation of HA-p53 in the presence of either ZNF or VHH-ZNF. Following the *in vitro* assays, we did not detect any SUMOylation for either condition, suggesting that VHH-ZNF is unable to promote efficient SUMOylation of HA-p53 (Figure 1E). In the absence of a GFP-tagged substrate, similar amounts of diSUMO2 accumulated in both conditions after 80 min (Figure 1F), suggesting that the fusion of ZNF to VHH does not strongly impact the SUMO E3 ligase activity of the ZNF moiety. Overall, these results indicate that the robust SUMOylation increase in the presence of VHH-ZNF is specific to GFP-fused substrate.

### The VHH-ZNF nanobody SUMOylates p53 *in trans* with similar efficacy and specificity for SUMO2/3 to the ZNF fusion tag

**Figure 2:**
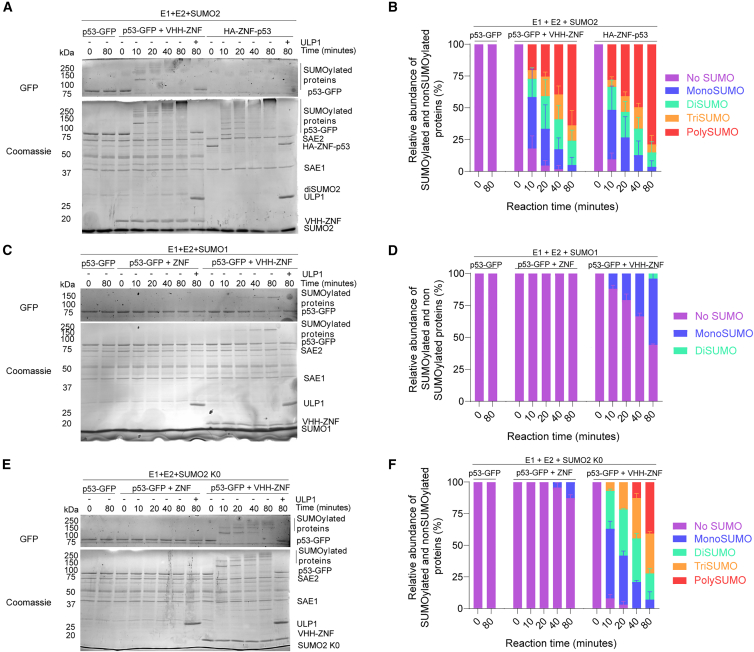
VHH-fused ZNF preferentially promotes SUMO2 poly/multiSUMOylation of GFP-fused p53 *in trans* (A, C, and E) *In vitro* assays comparing the SUMOylation of (A) p53-GFP+VHH-ZNF and HA-ZNF-p53 using SUMO2, (C) p53-GFP with ZNF or VHH-ZNF using SUMO1, or (E) using SUMO2 K0. (B, D, and F) Quantification of SUMOylation levels associated with the gels presented in (A), (C), and (E), respectively. Gels are a representative of three independent experiments (*N* = 3). All results are mean ± SD from *N* = 3.

Next, we wondered how the VHH-ZNF strategy, which promotes SUMOylation *in trans*, compares to the ZNF-fusion strategy, which promotes SUMOylation *in cis*.^21^ We thus compared the SUMOylation of p53-GFP with VHH-ZNF to that of HA-ZNF-p53 *in vitro*. Analysis of SUMOylation reactions performed *in vitro* reveals that both strategies promoted robust SUMOylation in a very similar manner (Figure 2A). Indeed, both substrates underwent rapid SUMOylation, with the unSUMOylated protein band becoming undetectable for both conditions after merely 20 min. Quantification of the reactions confirmed very similar kinetics of reaction (Figure 2B), underscoring the efficiency of the VHH-ZNF strategy.

We showed previously that the ZNF tag can promote SUMO1 modification of a fused substrate *in vitro*, although with much less efficiency than with SUMO2.^21^ To ensure that fusing ZNF to the nanobody does not affect this specificity, we tested the SUMOylation of p53-GFP with SUMO1 instead of SUMO2 in the presence of the VHH-ZNF or ZNF. After 80 min of reaction, about half of p53-GFP was monoSUMOylated in the presence of VHH-ZNF (Figures 2C and 2D) but not in the presence of unfused ZNF. This contrasts with our other assays, where complete modification of p53-GFP could be achieved with SUMO2 after only 10 min (Figure 1B) and suggests that VHH-ZNF is more efficient at promoting p53-GFP modification using SUMO2 rather than SUMO1. Overall, these results indicate that VHH-ZNF promotes protein SUMOylation with similar efficacy to the ZNF fusion strategy.

### PolySUMO2 chains on lysine K386 are the main type of SUMOylation promoted by VHH-ZNF

To distinguish between polySUMOylation and multiSUMOylation, we performed assays using a SUMO2 K0 variant of SUMO2 that cannot form chains, as all of its lysine residues were mutated to arginine residues. Similar to our previous findings,^21^ the use of this variant altered the SUMOylation patterns of p53-GFP. Indeed, although the band corresponding to unmodified p53-GFP disappeared in less than 10 min both with SUMO2 and SUMO2 K0, the formation of species containing more than three SUMOs was severely delayed for samples containing SUMO2 K0 (compare Figures 2A and 2B with Figures 2E and 2F). Consistent with SUMO2 K0 not being able to form SUMO chains, no diSUMO2 accumulated at the bottom of the gel (Figure 2E).

**Figure 3:**
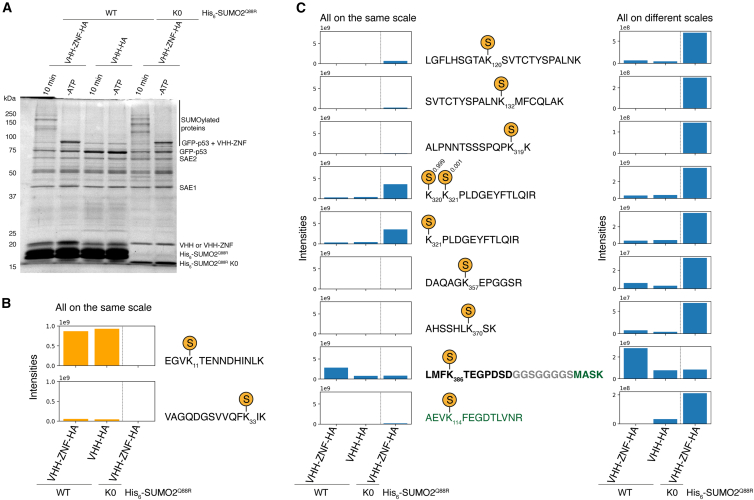
VHH-ZNF preferentially promotes polySUMOylation on K386 of p53 *in vitro* (A) SUMOylation reactions performed with p53-GFP, with either VHH-ZNF or ZNF, and His_6_-SUMO2^Q88R^ or His_6_-SUMO2^Q88R^ K0. Aliquots were taken before the reactions were started, then 5 mM ATP was added to start the reactions. After 10 min, a second aliquot was taken. Reactions were stopped by boiling for 5 min at 95°C. Aliquots were loaded on a 12% SDS-PAGE gel, which was stained with Coomassie blue. (B) Mass spectrometry analysis of the reaction in (A), showing the formation of a QQTGG adduct on His_6_-SUMO2^Q88R^ with p53-GFP, and ZNF or VHH-ZNF. The exact position of the adduct is shown. (C) Mass spectrometry analysis of the reaction in (A), showing the formation of a QQTGG adduct on p53-GFP, in the presence of ZNF or VHH-ZNF. The exact position of the adduct is shown. p53 residues are colored in black, the linker separating p53 and GFP is colored in gray, while GFP is colored in green. The peptide containing lysine 386 of p53 is in bold.

To identify the lysine targeted by SUMOylation, we analyzed the modification of p53-GFP with the mass spectrometry detectable His_6_-SUMO2^Q88R^ mutant after 10 min in the presence of VHH-ZNF or ZNF. We first confirmed that p53-GFP underwent SUMOylation after 10 min by migrating the samples on an SDS-PAGE gel (Figure 3A). Mass spectrometry identified QQTGG adducts on SUMO2 residues 11 and 33 with similar levels for ZNF and VHH-ZNF-containing reactions (Figure 3B). Consistent with previous results obtained with HA-ZNF-p53 fusion,^21^ these analyses also confirmed the SUMOylation of p53 at lysine 386, which is p53’s main acceptor site,^34^ with additional SUMOylation detected at positions 120, 320, 321, 357, and 370 (Figure 3C). SUMOylation at these positions was observed both in the presence of VHH-ZNF and the ZNF control, but with a higher relative SUMOylation of lysine 386 in the presence of VHH-ZNF, whereas SUMOylation of GFP at position 114 was observed only in the presence of VHH-ZNF (Figure 3C). The SUMOylation of p53-GFP by VHH-ZNF in the presence of a His_6_-SUMO2^Q88R^ K0 variant that cannot form chains allowed the detection of two additional sites of SUMOylation, lysine 132 and 319, within p53 (Figure 3C). The use of this SUMO variant also caused a reduction in the relative SUMOylation at lysine 386 compared to SUMOylation using His_6_-SUMO2^Q88R^, consistent with a prioritization of multiSUMOylation when chains cannot build on p53’s main acceptor site. In conclusion, these analyses are consistent with the interaction between p53-GFP and VHH-ZNF leading to the polySUMOylation of p53 on its Lys386 main acceptor site.

### Processive SUMOylation of p53-GFP by VHH-ZNF

**Figure 4:**
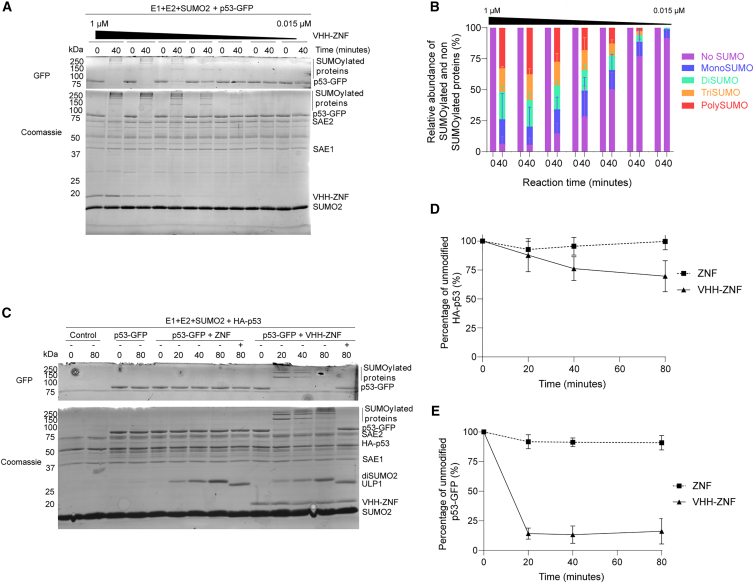
VHH-ZNF promotes SUMOylation in a concentration-dependent manner and can target other subunits of p53 (A) *In vitro* assay comparing the SUMOylation of p53-GFP in the presence of varying concentrations of VHH-ZNF, starting at 1 μM, and diluting 1:1 between each condition, with a 40-min reaction time. (B) Quantification of SUMOylation for the gel presented in (A). (C) *In vitro* assay comparing the SUMOylation of HA-p53 and p53-GFP in the presence of either ZNF or VHH-ZNF. (D and E) Percentage of unmodified HA-p53 (D) or p53-GFP (E) band intensity, in the presence of either ZNF or VHH-ZNF. Gels are a representative of three independent experiments (*N* = 3). All results are mean ± SD from *N* = 3.

While the ZNF fusion strategy is useful to study the impact of SUMOylation of specific proteins,^21^ this strategy cannot control the amplitude of modification because of the fixed ZNF-p53 stoichiometry imposed by the fusion. This raised the question of whether a modulation of VHH-ZNF concentrations could affect the SUMOylation of p53-GFP. To determine the impact of the VHH-ZNF concentration on p53-GFP’s SUMOylation, we kept the concentration of p53-GFP constant while progressively diluting VHH-ZNF. SUMOylation of p53-GFP at 80 min decreased in concordance with VHH-ZNF dilution (Figure 4A). Indeed, while the vast majority of p53-GFP undergoes SUMOylation at the 80 min time point with more than 50% multi/polySUMOylation, a mixture of unmodified and SUMO-modified species was observed in samples containing less VHH-ZNF (Figure 4B). The presence of poly/multi-SUMOylated bands even when a fraction of p53 is un-SUMOylated is consistent with a model whereby VHH-ZNF association to p53-GFP leads to processive SUMOylation, perhaps due to the tight binding of the interaction between VHH and GFP, with a measured K_D_ of ∼1 nM,^33^ or because SUMOylated p53 constitutes a better target for SUMOylation by VHH-ZNF than the unmodified protein. Interestingly, diSUMO2 accumulation appeared to fade away quicker than p53-GFP SUMOylation as a function of decreasing VHH-ZNF concentration (Figure 4A). Overall, these results indicate that modulating VHH-ZNF concentration can decrease non-specific SUMOylation in favor of processive substrate modification.

### VHH-ZNF recruitment by GFP-fused p53 facilitates the SUMOylation of unfused subunits of p53

As previously stated, p53 is mostly found in a tetrameric form, although subunit association is dynamic.^35^ As VHH-ZNF was unable to promote SUMOylation of HA-p53, we wondered if spiking the reaction with p53-GFP could lead to its SUMOylation through hetero-tetramer formation. Indeed, the band corresponding to unmodified HA-p53 partially faded in the presence of both p53-GFP and VHH-ZNF, with concomitant appearance of bands of higher molecular weight, suggesting HA-p53 SUMOylation by VHH-ZNF via the formation of hetero-oligomers that bring GFP in close proximity to HA-p53. Quantification of the HA-p53 protein band revealed a 25% reduction in intensity in the presence of VHH-ZNF compared to conditions containing unfused ZNF, in which HA-p53 remained largely unmodified, or to controls with co-transfected UPL1 (Figure 4D). This reduction followed a larger drop in the levels of unmodified p53-GFP, also observed only in the presence of VHH-ZNF (Figure 4E), consistent with efficient SUMOylation of p53-GFP. Taken together, these results are consistent with SUMOylation of HA-p53 by VHH-ZNF being dependent on the formation of p53 heterooligomers with p53-GFP.

### VHH-ZNF increases SUMOylation of GFP-tagged substrate in HEK293 cells

**Figure 5:**
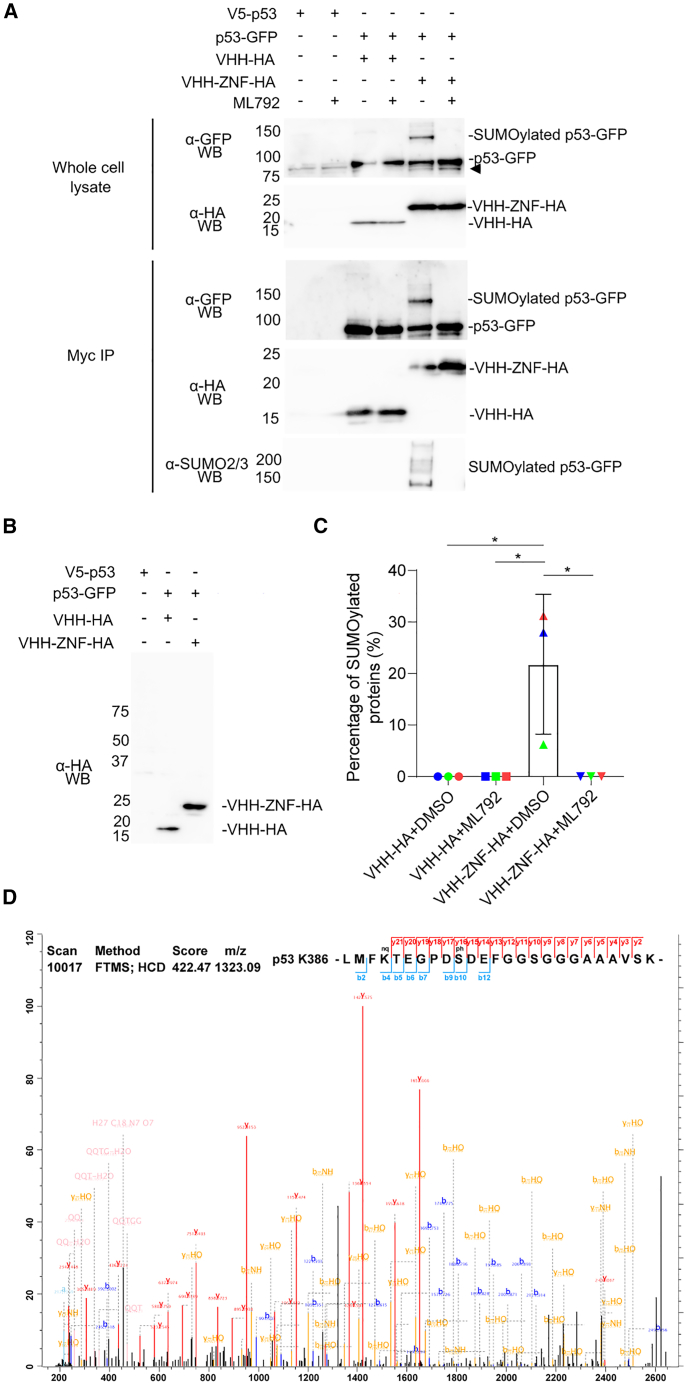
VHH-ZNF increases SUMOylation of GFP-fused p53 on lysine 386 in HEK293 cells (A) Immunoblot analysis of whole cell lysate or immunoprecipitated p53-GFP-Myc, in the presence of either VHH-ZNF-HA or VHH-HA. HEK293 cells were transfected with pcDNA3.1 vectors expressing V5-p53, p53-GFP-Myc-P2A-VHH-HA, or p53-GFP-Myc-P2A-VHH-ZNF-HA for 48 h. Cells were treated for 4 h with 5 μM ML792 or control (0.01% DMSO), then lysed. Immunoprecipitations were performed using an anti-Myc antibody overnight at 4°C. Beads were then washed using lysis buffer, and elution was performed with Laemmli buffer. All proteins were separated on a 12% SDS-PAGE gel, except samples detected with an anti-SUMO2/3 antibody, which were separated on a 7.5% SDS-PAGE gel. Immunoblots were performed on whole cell lysates and immunoprecipitated proteins using anti-GFP, anti-HA or anti-SUMO2/3 antibodies. The membranes used for anti-GFP and anti-HA whole-cell lysates and immunoprecipitated proteins were cut at 37 kDa in order to use both antibodies on the same membrane. The triangle indicates a nonspecific band that is also present in samples lacking HA-tagged proteins. (B) Samples presented in panel (A) were separated on a 12% SDS-PAGE gel. Immunoblot on whole cell lysate was performed using an anti-HA antibody. (C) The percentage of SUMOylated proteins was obtained by dividing the band intensity corresponding to SUMOylated species by the band intensity corresponding to unmodified p53-GFP obtained from the anti-GFP immunoblot of the immunoprecipitation, in (A). Statistical analysis was performed using a multiple comparison two-way ANOVA followed by a Tukey test, ∗*p* < 0.05. (D) Mass spectrum of p53-GFP-myc in the presence of VHH-ZNF-HA transfected in HEK293 cells stably expressing His6-SUMO3^Q87R/Q88N^ and showing SUMOylation on lysine 386 and phosphorylation on Ser392. All results are mean ± SD from *N* = 3.

We previously showed that fusing the ZNF tag to a substrate could increase its SUMOylation in HEK293 cells.^21^ To find out whether VHH-ZNF could be as efficient in this model, we generated a plasmid encoding p53-GFP-myc separated from either VHH-ZNF-HA or VHH-HA by a self-cleaving P2A peptide to ensure that every transfected cell would express both p53-GFP-myc and VHH-(ZNF)-HA. An anti-GFP immunoblot of whole cell lysates confirmed that the additional presence of ZNF in VHH-HA did not impact p53-GFP-myc protein expression (Figure 5A). A band corresponding to SUMOylated p53-GFP-myc was detected on the anti-GFP immunoblot of whole cell lysates in conditions with VHH-ZNF-HA, but not with VHH-HA, even though both nanobodies were present in similar amounts, as revealed using an anti-HA antibody (Figure 5A). We verified that P2A self-cleavage was mostly complete, as only one intense protein band appeared in each lane at the expected molecular weight for VHH-ZNF-HA and VHH-HA when immunoblotting the whole cell lysate with an anti-HA antibody on the entirety of the membrane (Figure 5B). Consistent with observations made on whole-cell lysates, a band corresponding to SUMOylated p53-GFP-myc was only detected in the presence of VHH-ZNF-HA in immunoprecipitations performed using an anti-myc antibody (Figure 5A). Quantification further indicated that around 20% of the p53-GFP-myc protein was modified (Figure 5C). An anti-HA immunoblot on the bottom part of the gel revealed that both nanobodies were also recovered by immunoprecipitation (Figure 5A), in agreement with their strong interaction with GFP.^33^

Consistent with the results obtained from the anti-GFP immunoblot, an anti-SUMO2/3 antibody detected strong poly/multiSUMOylation of p53-GFP-myc only in the presence of VHH-ZNF-HA in the anti-myc immunoprecipitates. Upon cell treatment with the SUMO E1 inhibitor ML792, both the putative monoSUMOylated band visible on the anti-GFP immunoblot and the poly/multiSUMOylated bands on the anti-SUMO2/3 immunoblot disappeared, confirming that the bands migrating at a higher apparent molecular weight than p53-GFP-myc indeed correspond to SUMOylated species. Taken together, these results indicate that VHH-ZNF promotes the efficient SUMOylation of a GFP-fused substrate in transfected HEK293 cells.

### p53 is SUMOylated at the canonical K386 site in HEK293 cells in a VHH-ZNF-dependent manner

We showed through *in vitro* experiments that VHH-ZNF mainly targeted lysine 386 of p53-GFP, although other lysine residues were also modified. To determine the specificity of VHH-ZNF in cells, we transfected our expression vector for p53-GFP-myc and either VHH-ZNF-HA or VHH-HA separated by a self-cleaving P2A peptide in HEK293 cells stably expressing His_6_-SUMO3^Q87R/Q88N^. Consistent with our previous analysis of HA-ZNF-p53 fusion,^21^ only SUMOylation at lysine 386 could be identified by mass spectrometry (Figure 5D). Importantly, this modified site could only be detected for p53-GFP in the presence of VHH-ZNF-HA, but not in the VHH-HA control. While our *in vitro* mapping identifies K386 as the main acceptor site, additional low-abundance SUMOylation events can be detected at other lysine residues (K120, K320/321, K357, and K370), and further sites emerge when SUMO2 chain formation is disabled (SUMO2(K0)), consistent with a shift toward multi-SUMOylation. In cells, however, SUMOylation is constrained by substrate conformation/complex assembly and competing interactions, and is dynamically edited by SUMO proteases; thus, secondary-site modification is expected to be transient/low stoichiometry and may be rapidly removed or processed *in vivo*. Together with lower effective substrate abundance in cellular extracts, these factors likely explain why only the dominant K386 modification is detected in our in-cell enrichment/MS workflow. Consistent with our previous findings,^21^ serine 392 was phosphorylated in the context of the p53 peptides SUMOylated at lysine 386, suggesting an interplay between both PTMs. The absence of detectable SUMOylation in cells for the GFP and myc tags is consistent with the greater accessibility or propensity for SUMOylation of the main acceptor site in p53.

### SUMOylated p53 accumulates in nuclear foci

**Figure 6:**
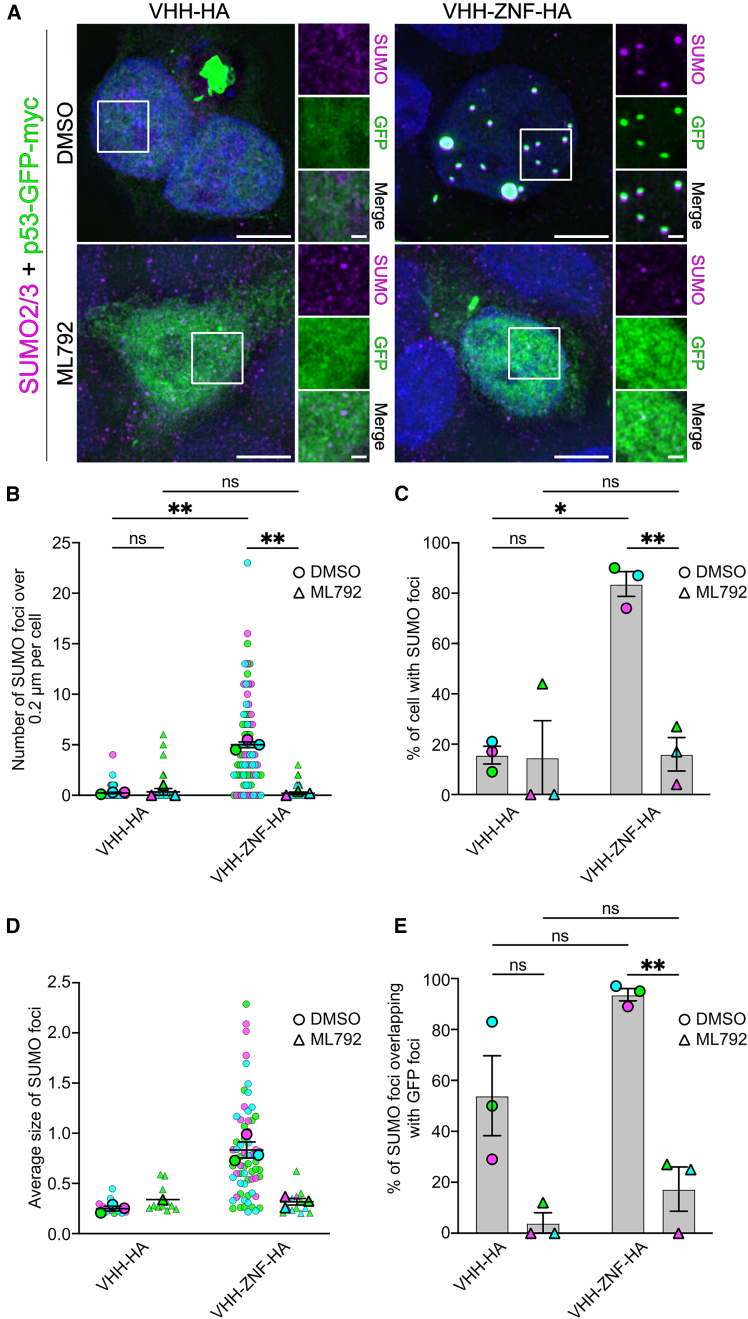
SUMOylated p53 forms nuclear foci in HEK293 cells (A) HEK293 cells were transfected with pcDNA3.1 vectors expressing p53-GFP-Myc-P2A-VHH-HA or p53-GFP-Myc-P2A-VHH-ZNF-HA. 48 h post-transfection, cells were treated with 5 μM ML792 or vehicle (0.01% DMSO) for 4 h. Cells were fixed, permeabilized, and stained using anti-SUMO2/3 primary antibody against endogenous protein. Representative images of three different experiments (*N* = 3) are shown. Scale bars indicate 5 μm for whole cell image and 1 μm for higher magnification. (B–E) Quantifications were obtained from the experiments presented in (A) and present (B) number of SUMO foci larger than 0.2 μm per cell, (C) percentage of cells with at least 1 SUMO foci, (D) average size of SUMO foci and (E) percentage of SUMO2/3 foci overlapping with GFP foci. All results are mean ± SEM from *N* = 3. Larger points represent the mean of an independent experiment (*N* = 3). Smaller points represent each individual (B) cell or (D) foci analyzed (DMSO + VHH-HA, *n* = 71 cells/15 foci; ML792 + VHH-HA, *n* = 73 cells/26 foci; DMSO + VHH-ZNF-HA, *n* = 75 cells/370 foci; ML792 + VHH-HA, *n* = 73 cells/16 foci). ∗*p* < 0.05; ∗∗*p* < 0.01.

The expression of p53 fused with GFP allowed us to determine its subcellular localization and any changes promoted by SUMOylation using VHH-ZNF-HA. We used fluorescence microscopy to directly detect p53-GFP, while SUMO2/3 was detected through immunostaining (Figure 6A). While p53-GFP mostly had a diffuse localization in the nucleus in the presence of VHH-HA, it was mostly found as large nuclear foci in the nucleus the presence of VHH-ZNF-HA (Figure 6A) with overlapping SUMO and GFP signal. Quantifications of the deconvoluted images further indicated that there were significantly more SUMO foci with a diameter of more than 0.2 μm per cell in conditions with VHH-ZNF-HA than with VHH-HA (Figure 6B). Upon ML792 treatment, the amount of SUMO foci per cell significantly diminished for VHH-ZNF-HA compared to the untreated cells, reaching levels comparable to those observed with VHH-HA (Figures 6A and 6B). Consistent with these results, the percentage of cells with SUMO foci was also significantly higher for cells expressing VHH-ZNF-HA, while treatment with ML792 significantly diminished this percentage to a level comparable to VHH-HA cells (Figure 6C). The average size of SUMO foci followed the same pattern, as SUMO foci were larger in the presence of VHH-ZNF-HA compared to VHH-HA, while ML792 treatment reestablished sizes comparable to those in conditions with VHH-HA (Figure 6D). Taken together, these results indicate that VHH-ZNF-HA promotes the formation of SUMO foci in the nucleus, in a SUMOylation-dependent manner. We then assessed the overlap between SUMO foci and the p53-GFP signal and found that 94% of SUMO foci overlapped with the p53-GFP signal in the presence of VHH-ZNF-HA, whereas this percentage was around 54% in the presence of VHH-HA (Figure 6E). These analyses, however, must be nuanced by the fact that fewer foci are detected in the presence of VHH-HA (15 foci) than in the presence of VHH-ZNF-HA (370 foci). Importantly, treatment with ML792 significantly decreased this overlap for the VHH-ZNF-HA condition (Figure 6E), thus suggesting that the p53-GFP foci colocalize with SUMO in a SUMOylation-dependent manner.

We then repeated this experiment with HA-immunostaining in lieu of endogenous SUMO2/3 immunostaining (Figure S1A). While the same nuclear relocalization of p53-GFP was observed in a ZNF-dependent manner, these analyses also indicated a more cytoplasmic localization for VHH-HA whereas VHH-ZNF-HA was mainly found in the nucleus (Figure S1A). Finally, to confirm that the observed phenotypes were dependent on ZNF and not biased by the interaction between GFP and VHH, we transfected HEK293 cells with HA-ZNF-p53 or HA-p53 and immunostained the cells using an anti-HA antibody and an anti-SUMO2/3 antibody. Interestingly, and similar to what we observed with the nanobody, HA-ZNF-p53 was mostly found in nuclear foci that overlapped with SUMO, while HA-p53 was mostly diffused in the nucleus (Figure S1B). This suggests that ZNF, not GFP or VHH, promotes p53 localization to SUMO foci. Taken together, these results indicate that VHH-ZNF-HA promotes the formation of SUMO foci in the nucleus of HEK293 cells expressing p53-GFP-myc in a SUMOylation-dependent manner.

### SUMOylation of p53 increases colocalization with PML and other nuclear bodies

**Figure 7:**
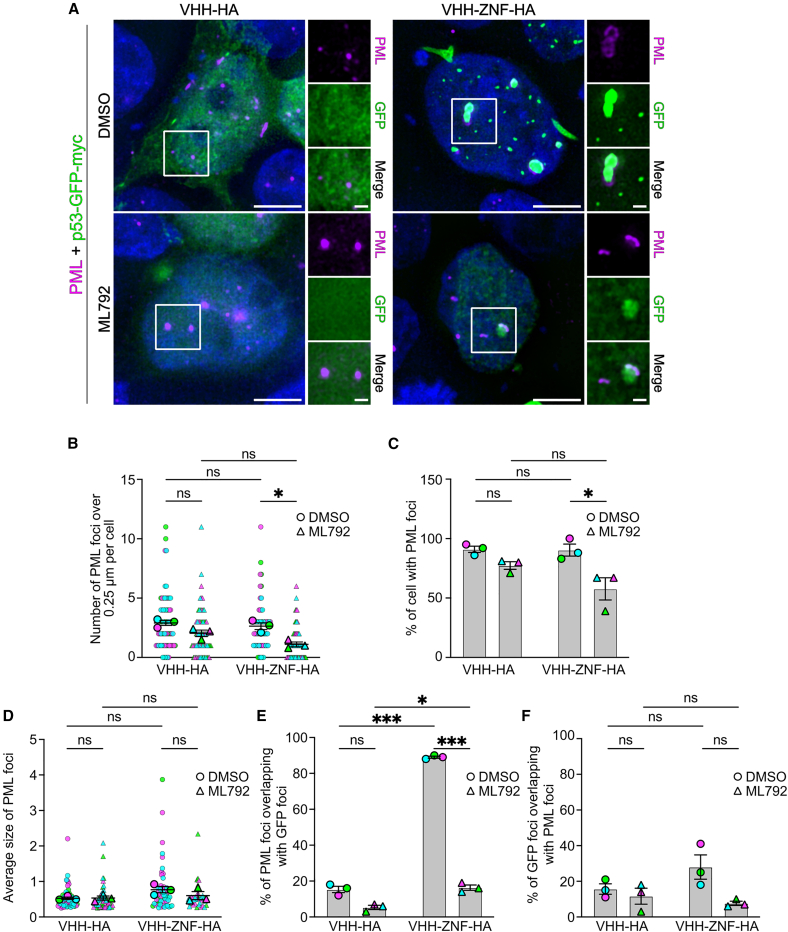
SUMOylation of p53 increases its subcellular localization to PML-NBs (A) HEK293 cells were transfected with pcDNA3.1 vectors expressing p53-GFP-Myc-P2A-VHH-HA or p53-GFP-Myc-P2A-VHH-ZNF-HA. 48 h post-transfection, cells were treated with 5 μM ML792 or vehicle (0.01% DMSO) for 4 h. Cells were fixed, permeabilized, and stained using anti-PML primary antibody against endogenous protein. Representative images of three different experiments (*N* = 3). Scale bars indicate 5 μm for whole cell image and 1 μm for higher magnification. (B–E) Quantifications were obtained from the experiments presented at (A) and present the (B) number of PML foci larger than 0.25 μm per cell, (C) percentage of cells with at least 1 PML foci, (D) average size of PML foci, (E) percentage of PML foci overlapping with GFP foci and (F) percentage of GFP signal overlapping with PML signal. All results are mean ± SEM from *N* = 3. (B and D) Larger points represent the mean of an independent experiment (*N* = 3). Smaller points represent each individual (B) cell or (D) foci analyzed (DMSO + VHH-HA, *n* = 69 cells/202 foci; ML792 + VHH-HA, *n* = 65 cells/131 foci; DMSO + VHH-ZNF-HA, *n* = 63 cells/165 foci; ML792 + VHH-HA, *n* = 65 cells/72 foci). ns = not significant, ∗*p* < 0.05; ∗∗∗*p* < 0.001 using two-way RM-ANOVA with uncorrected Fisher’s LSD multiple comparisons.

Since p53 was mostly found in large nuclear foci in a VHH-ZNF-HA and SUMOylation-dependent manner, and since PML NBs are known to constitute hubs for SUMOylated proteins,^27^^,^^28^^,^^29^ we verified whether p53-GFP localizes within PML. Immunostaining using anti-PML antibodies revealed nuclear foci in the deconvoluted images (Figure 7A). The p53-GFP foci with VHH-ZNF-HA and DMSO coincided with PML bodies, although the overlap was not perfect. Instead, PML and p53-GFP appeared slightly juxtaposed, as previously reported for p53.^36^

No significant differences in the number of PML bodies per cell were found between VHH-HA and VHH-ZNF-HA conditions, although treatment with ML792 did decrease the number of PML bodies per cell in the latter condition (Figure 7B). Similar observations were made for the percentage of cells with PML NBs, as no significant difference was observed between VHH-HA and VHH-ZNF-HA (Figure 7C). Once again, ML792 treatment significantly decreased the number of cells with PML foci in the presence of VHH-ZNF-HA (Figure 7C). Lastly, the presence of VHH-ZNF-HA did not impact the size of PML bodies compared to the VHH-HA condition, nor did ML792 treatment (Figure 7D). We thus conclude that VHH-ZNF-HA does not have a strong impact on the size or amount of PML bodies.

To determine if SUMOylated p53 localizes to PML bodies, we quantified the overlap between the PML signal and the p53-GFP foci. Quantification shows that 89% of PML foci overlap with p53-GFP in the presence of VHH-ZNF-HA, which is significantly more than the 16% that overlap in the presence of VHH-HA (Figure 7E). Furthermore, ML792 treatment strongly reduced this overlap, bringing the levels close to those in the conditions with VHH-HA. Intriguingly, no statistical difference was observed for the reciprocal overlap of GFP foci on PML signal in every condition and where overlap never exceeded 30% (Figure 7F). These results suggest that the SUMOylation of p53 promotes its partial relocalization to PML bodies but possibly also to other NBs or structures.

**Figure 8:**
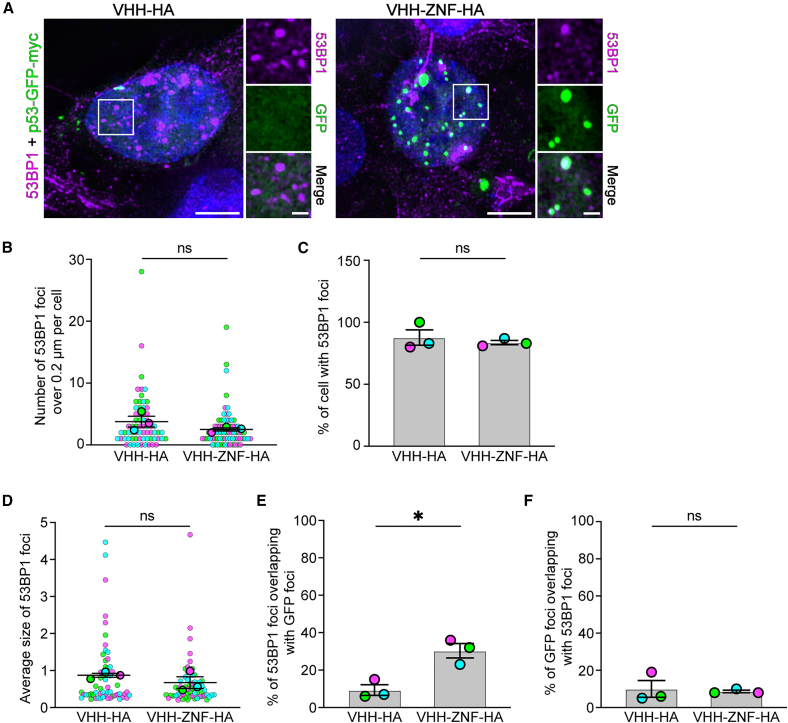
SUMOylation of p53 increases its subcellular localization to 53BP1-NBs (A) Plasmids encoding p53-GFP with either VHH-HA or VHH-ZNF-HA separated by a self-cleaving peptide P2A were transfected in HEK293 cells. Cells were fixed, permeabilized, and stained using anti-p53BP1 primary antibody against endogenous protein. Representative images of three different experiments (*N* = 3). Scale bars indicate 5 μm for whole cell image and 1 μm for higher magnification. (B–F) Quantifications were obtained from the experiments presented at (A) and present the (B) number of p53BP1 foci larger than 0.2 μm per cell, (C) percentage of cells with at least 1 p53BP1 foci, (D) average size of p53BP1 foci, (E) percentage of p53BP1 foci overlapping with GFP foci and (F) percentage of GFP signal overlapping with p53BP1 signal. All results are mean ± SEM from *N* = 3. (B and D) Larger points represent the mean of an independent experiment (*N* = 3). Smaller points represent each individual (B) cell or (D) foci analyzed (VHH-HA, *n* = 66 cells/240 foci; VHH-ZNF-HA, *n* = 73 cells/187 foci). ns = not significant, ∗*p* < 0.05 using two-tailed paired *t* test.

Finally, we investigated whether p53 could localize to other NBs following its SUMOylation. We focused on p53-binding protein 1 (53BP1), a protein known to interact with p53,^37^ involved in the DNA damage response,^38^ and found in NBs notably around DNA double-strand breaks.^39^ Immunostaining using an anti-53BP1 antibody revealed a mixed pattern of diffuse staining and distinct foci within nuclei (Figure 8A). Intriguingly, in the presence of VHH-ZNF-HA, some of the p53-GFP foci overlapped with 53BP1foci. No significant difference was found when counting the number of foci per cell (Figure 8B), although a much smaller number of 53BP1 nuclear foci were detected compared to PML NBs. Importantly, no significant difference was observed between VHH-HA and VHH-ZNF-HA for the number of cells with 53BP1 NBs (Figure 8C). Similar observations were made on the average foci size for both conditions (Figure 8D).

To determine if SUMOylated p53-GFP localizes in 53BP1 NBs, we quantified the overlap between the 53BP1 signal and the p53-GFP foci. Quantifications show that 30% of the 53BP1 foci overlap GFP signal in the presence of VHH-ZNF-HA, while less than 9% did with VHH-HA (Figure 8E). Once again, no significant difference was detected when overlapping the GFP signal with 53BP1 foci, confirming the observations made with PML. Overall, these results suggest that, although SUMOylated p53 can localize at other NBs such as 53BP1 NBs, its main target appears to be PML NBs.

## Discussion

This research focused on the elaboration of a tool capable of promoting the SUMOylation of GFP-fused substrates *in trans* to study SUMOylation-dependent changes in protein localization. To achieve this, we fused a VHH nanobody that specifically recognizes GFP to a ZNF SUMOylation tag that we previously developed.^21^ Through *in vitro* assays, we demonstrated the capacity of VHH-ZNF to increase the SUMOylation of p53-GFP, but not HA-p53 alone, showcasing its specificity for GFP-tagged proteins. Furthermore, the SUMOylation levels obtained with VHH-ZNF were comparable to those obtained with HA-ZNF-p53, which promotes SUMOylation *in cis*, in accordance with the tight association between GFP and VHH.^33^ The specificity of ZNF for SUMO2 was retained when fused to VHH, as VHH-ZNF showed less activity with SUMO1. Mass spectrometry experiments suggested that VHH-ZNF induced SUMOylation of p53 at its endogenous sites, most notably at its main site lysine 386.^34^ We further showed it is possible to control the amount of SUMOylation of p53 by reducing the concentrations of VHH-ZNF nanobody and provided evidence that VHH-ZNF also targets the other subunits of p53, as HA-p53, which is unmodified in the presence of VHH-ZNF, becomes SUMOylated in the presence of both p53-GFP and VHH-ZNF, at least *in vitro*. We confirmed that VHH-ZNF is capable of SUMOylating GFP-tagged proteins in cells, as we showed a SUMOylation increase of p53-GFP in the presence of VHH-ZNF through immunoprecipitation and immunoblotting. This increase is dependent on the presence of ZNF, as VHH-HA was unable to promote SUMOylation of p53-GFP. Ultimately, we showed that SUMOylated p53 mainly localizes in nuclear foci overlapping with SUMO, unlike the p53 control, which has a diffuse location in the nucleus. Notably, VHH-ZNF greatly increases the overlap of PML NB with p53-GFP, suggesting that p53 SUMOylation promotes its recruitment to these structures as well as, to a lesser extent, other NBs such as 53BP1 NBs. Overall, these results confirm that VHH-ZNF promotes SUMOylation of GFP-tagged substrates *in trans*, and that this tool can be used to study the effects of protein SUMOylation on protein localization.

Comparing our nanobody-based method (in *trans*) with our previous linear fusion approach^21^ further revealed that both effectively promoted poly/multiSUMOylation (Figures 2A and 2B) while retaining specificity for SUMO2 *in vitro* (Figures 1B and 2C). Importantly, titrating VHH-ZNF concentrations allowed fine-tuning of diSUMO2 levels without affecting poly/multiSUMOylation (Figures 4A and 4B), highlighting the importance of adjusting stoichiometry to minimize non-specific SUMOylation.

In cells, while immunofluorescence showed that VHH-HA mainly localized in the cytoplasm, VHH-ZNF-HA was mostly nuclear (Figure S1A). Importantly, our results indicated that both SUMOylated and unmodified p53-GFP remained in the nucleus (Figures 6A, 7A, and 8A). SUMOylated p53-GFP was, however, found in nuclear SUMO foci partially overlapping with PML NBs, contrarily to the diffuse location of unmodified p53-GFP. The fact that about 89% of PML-NBs colocalized with p53-GFP in the presence of VHH-ZNF-HA (Figure 7E), whereas only 28% of p53-GFP colocalized with PML NBs (Figure 7F) suggests that SUMOylated p53 may be directed to other nuclear structures, in addition to PML-NBs, such as 53BP1 NBs (Figure 8). Interestingly, VHH-ZNF did not promote the formation of PML NBs (Figure 7B), nor did it increase their size (Figure 7D), implying that p53-GFP SUMOylation directly promotes its localization to PML-NBs, without altering the formation of these structures. It is tempting to speculate that PML localization affects p53 transcription activity through changes in protein interactions. p53 was previously reported to localize at PML NBs,^31^ although the importance of SUMO in this process was disputed.^32^ Notably, p53 is known to localize to PML upon certain cellular stress, where it interacts with other proteins, such as HIPK2,^40^ SIRT1,^41^ and CBP/p300,^42^ leading to either apoptosis or the inactivation of p53, depending on whether DNA damage is reversible.^43^^,^^44^ Furthermore, PML-NBs can lead to p53 cellular accumulation, either by sequestering MDM2, the main ubiquitin E3 responsible for degrading p53,^45^ to nucleoli,^46^ or by promoting the phosphorylation of p53 by CK1, protecting it from MDM2-mediated degradation.^47^ If our results reveal that nanobody-induced SUMOylation of p53 modifies its localization, the exact consequences of this change on p53 transcriptional activity remain to be studied in detail.

While PML NBs colocalization with p53-GFP did increase in the presence of VHH-ZNF-HA (Figure 7E), p53-GFP colocalization with PML NBs did not increase significantly (Figure 7F), suggesting that SUMOylated p53 could localize with other NBs. Notably, we showed an increased overlap of 53BP1 NBs, which mostly form around double-strand breaks,^39^ with p53-GFP (Figure 8E). Other potential NBs where SUMOylated p53 could perhaps localize include Polycomb group (PcG) bodies, nuclear hubs of epigenetic gene silencing,^48^ which are also regulated by SUMOylation.^49^ Alternative locations include paraspeckles, RNA-protein bodies which play a role in stress response,^50^ Cajal bodies,^51^ or even liquid-liquid phase separation.^52^

Overall, our approach has a potential to reveal more SUMOylation-induced changes in protein localization. Similar to p53, we predict that other proteins could also localize to PML-NBs once SUMOylated, thus leading to possible changes in interacting partners. In PML case, it was reported that roughly 45% of the PML-NBs proteome is associated with senescence and the aging process.^53^ Numerous transcription factors localize to PML-NBs, such as p53,^31^^,^^32^ and DAXX^25^ and PML-NBs were also shown to interact with chromatin.^54^ In this context, the use of our tool could help exploring the SUMOylation-dependent links between PML-NBs, transcription and stresses.

Our results finally revealed that VHH-ZNF targeted non-GFP-tagged p53 if mixed with GFP-tagged p53 (Figures 4C and 4D), suggesting proximity-based SUMOylation of other subunits of the tetramer. If the SUMOylation of other p53 subunits is detectable *in vitro*, it is, however, unclear if this is sufficient to trigger the SUMOylation of other proteins in cells. Future proteomics studies may provide a clearer view of whether p53 subunits, p53 interactors, or even neighboring proteins may be targeted. The relatively tight interaction between p53 subunits—with a dissociation constant of ∼0.1–1 μM for p53 tetramer and ∼1 nM for p53 dimers^55^—suggests that tight interactors may be preferentially modified. If proximity-based SUMOylation is confirmed in cells, it could highlight a potential use for our approach to promote SUMOylation of strong interactors. For example, we envision that VHH-ZNF could be used to facilitate the identification of SUMO-dependent interactors through SUMO-ID^56^ or other proteomic approaches.

Recently, other PTMs have been successfully implemented in modern biotechnologies to achieve specific goals. One example is ubiquitin with the proteolysis-targeting chimeras (PROTACs),^57^ small heterofunctional compounds that are capable of binding specific substrates, promoting their ubiquitination *in trans*, ultimately leading to protein degradation. Unlike ubiquitin, SUMOylation did not have attractive handles to promote SUMOylation. As we proved that *in trans* targeted SUMOylation is feasible, ZNF could be implemented in a SUTACs approach,^58^ inspired by PROTACs, that could instead modulate transcription factor activity and protein localization. For instance, the fusion of ZNF to known compounds recognizing α-Synuclein,^59^ whose aggregation has been implicated in Parkinson’s disease,^60^ could help enhance its SUMOylation and perhaps restrict its aggregation propensity.^61^

### Limitations of the study

The results were obtained using a single cell line, HEK293, and a single substrate, p53, as a proof of principle. This study also did not determine the consequences of nanobody-induced SUMOylation on p53 functions such as its transcriptional activity.

## Resource availability

### Lead contact

Requests for further information and resources should be directed to and will be fulfilled by the lead contact, Laurent Cappadocia (cappadocia.laurent@uqam.ca).

### Materials availability

All unique/stable reagents generated in this study are available from the lead contact without restriction.

### Data and code availability

• Data: The proteomics data analyzed in this study has been deposited to the ProteomeXchange Consortium (http://proteomecentral.proteomexchange.org) via the PRIDE partner repository under accession PXD069854.
• Code: This paper does not report original codes
• All other items: Any additional information required to reanalyse the data reported in this paper are available from the lead contact upon request.

## Acknowledgments

This work was supported by a 10.13039/501100000038Natural Sciences and Engineering Research Council of Canada Discovery grant to L.C. (RGPIN-2019-06807) and a *Fonds de recherche du Québec—Nature et technologies sector* (10.13039/501100003151FRQNT) Team Research Project grant to P.T., L.C., and S.M. (award number 345435). The *Center d’Excellence en Recherche sur les Maladies Orphelines—Fondation Courtois* (10.13039/501100021106CERMO-FC) funded part of this study via its program Acceleration (to L.C. and M.P.L.; 2020) and a graduate PhD scholarship from FRQNT was awarded to A.Y.B. (https://doi.org/10.69777/317082) and V.C.C. (https://doi.org/10.69777/326578). A graduate Master’s Research Scholarship from FRQNT (https://doi.org/10.69777/332296) and a CGS M (Canada Graduate Scholarship—Master’s) were awarded to J.P. The *Société de recherche sur le cancer* awarded a Ph.D. graduate scholarship to A.V. (2025). We would like to thank James Omichinski and its group for discussion and reagents.

## Author contributions

Conceptualization and methodology, A.Y.B., V.C.C., J.P., C.L., A.J.I.V., S.M., P.T., M.P.L., and L.C.; investigation, validation and formal analysis, A.Y.B., V.C.C., and C.L.; resources, J.P., S.M., P.T., M.P.L., and L.C.; visualization, A.Y.B., V.C.C., and C.L.; writing—original draft preparation, A.Y.B.; writing—review and editing, A.Y.B., V.C.C., J.P., C.L., A.J.I.V., S.M., P.T., M.P.L., and L.C.; supervision, S.M., P.T., M.P.L., and L.C.; project administration, M.P.L. and L.C.; funding acquisition, P.T., S.M., and L.C. All authors have read and agreed to the published version of the manuscript.

## Declaration of interests

The authors declare no competing interests.

## STAR★Methods

### Key resources table

**Table:** 

REAGENT or RESOURCE	SOURCE	IDENTIFIER
**Antibodies**		

Rabbit anti-SUMO2/3	Sigma	Cat#S9571; RRID: AB_796170
Rabbit anti-HA	BioLegend	Cat#902302; RRID: AB_256019
Mouse anti-HA	BioLegend	Cat#901503; RRID: AB_2629623
Mouse anti-myc	New England Biolabs	Cat#2276S; RRID: AB_331783
Rabbit anti-PML	ThermoFisher	Cat#A301-167A-M; RRID: AB_2779722
Rabbit anti-GFP	Invitrogen	Cat#A-6455; RRID: AB_221570
Rabbit anti-53BP1	ThermoFisher	Cat#A300-272A; RRID: AB_185520
HRP-conjugated Donkey anti-rabbit	Cell Signaling	Cat#7074S; RRID: AB_2099233
AlexaFluor 647-conjugated Goat anti-Rabbit	ThermoFisher	Cat#A-21245; AB_2535813
AlexaFluor 488-conjugated Goat anti-Mouse	ThermoFisher	Cat#A-11017; RRID: AB_253408
AlexaFluor 488-conjugated Goat anti-Rabbit	ThermoFisher	Cat#A-11070; RRID: AB_2534114

**Bacterial and virus strains**		

BL21DE3 Gold Codon Plus RIL	Novagen	N/A
DH5 alpha	Novagen	N/A

**Chemicals, peptides, and recombinant proteins**		

SAE1/SAE2	Bouchard et al.^21^	N/A
Ubc9	Bouchard et al.^21^	N/A
SUMO1	Bouchard et al.^21^	N/A
SUMO2	Bouchard et al.^21^	N/A
SUMO2 K0	Bouchard et al.^21^	N/A
SUMO2 Q88R	Bouchard et al.^21^	N/A
SUMO2 K0 Q88R	Bouchard et al.^21^	N/A
ULP1	Bouchard et al.^21^	N/A
HA-p53	Bouchard et al.^21^	N/A
p53-GFP	This paper	N/A
VHH-ZNF	This paper	N/A
ZNF451^25−52^	Bouchard et al.^21^	N/A
Dulbecco’s Modified Eagle Medium	Wisent	Cat#319-016 CL
Fetal Bovine Serum	Wisent	Cat#090-150
Polyethylenimine	PolySciences	Cat#23966-1
ML792	Cayman Chemical	Cat#36820
protease inhibitor cocktail	Selleckchem	Cat#B14002
N-Ethylmaleimide	Sigma-Aldrich	Cat#04259-5G

**Deposited data**		

Mass spectrometry data	This paper	PRIDE repository: PXD069854

**Experimental models: cell lines**		

HEK293 cells	American Type Culture Collection (ATCC)	N/A
HEK293 cells stably expressing 6xHis-SUMO3-Q87R/Q88N	Li et al.^11^	N/A

**Oligonucleotides**		

See Figure S2	This Paper	N/A

**Recombinant DNA**		

pET28b/SAE2^1−550^	Lois et al.^62^	N/A
pET11c/SAE1	Lois et al.^62^	N/A
pET28b/ULP1^403−621^	Mossessova and Lima.^63^	N/A
pRSFDuet/Ubc9	Bouchard et al.^21^	N/A
pRSFDuet/SUMO1^1−97^	Bouchard et al.^21^	N/A
pRSFDuet/SUMO2^1−93^	Bouchard et al.^21^	N/A
pRSFDuet/SUMO2 K0	Bouchard et al.^21^	N/A
pRSFDuet/SUMO2 Q88R	Bouchard et al.^21^	N/A
pRSFDuet/SUMO2 K0 Q88R	Bouchard et al.^21^	N/A
pRSFDuet/ZNF451^24−55^	Bouchard et al.^21^	N/A
pTRX/HA-p53	Bouchard et al.^21^	N/A
pTRX/HA-ZNF-p53	Bouchard et al.^21^	N/A
pTRX/VHH-ZNF	This paper	N/A
pTRX/p53-GFP	This paper	N/A
pcDNA3.1/HA-p53	Bouchard et al.^21^	N/A
pcDNA3.1/V5-p53	Bouchard et al.^21^	N/A
pcDNA3.1/HA-ZNF-p53	Bouchard et al.^21^	N/A
pcDNA3.1/p53-GFP-myc-P2A-vhhGFP4-ZNF-HA	This paper	N/A
pcDNA3.1/p53-GFP-myc-P2A-vhhGFP4-HA	This paper	N/A
pcDNA3/NSlmb-vhhGFP4	Caussinus et al.^64^	Addgene # 35579
pcDNA3/p53	Loughery et al.^65^	Addgene # 69003
pcDNA3.1 (+) (Invitrogen)	Invitrogen	Cat#V79020
pTRX	Bouchard et al.^21^	N/A

**Software and algorithms**		

Fiji	Schindelin et al.^66^	https://imagej.net/software/fiji/
ImageLab	BioRad	https://www.bio-rad.com/en-be/product/image-lab-software?ID=KRE6P5E8Z
MaxQuant	Cox et al.^67^	https://maxquant.org/
Prism 8.0.1	GraphPad	http://www.graphpad.com/

### Experimental model and study participant details

HEK293 cells heterozygous for p53 R280S were cultured in 5% CO_2_-containing humidified air at 37 °C. Genders and sexes were not taken in consideration for this research. Cells obtained by the Mader laboratory and were authenticated by STR (short tandem repeat) profiling on February 24, 2026 and regularly tested for mycoplasma contamination.

### Method details

#### Reagents

HEK293 cells were cultured using Dulbecco’s Modified Eagle Medium (DMEM, Wisent, Cat. No. 319-016 CL) supplemented with 10% Fetal Bovine Serum (FBS, Wisent, Cat. No. 090–150). For transfection, Polyethylenimine (PEI) was purchased from PolySciences (Cat. No. 23966-1). For cell treatment, Sulfamic acid, [(1R,2S,4R)-4-[[5-[[1-[(3-bromophenyl)methyl]-1H-pyrazol-3-yl]carbonyl]-4-pyrimidinyl]amino]-2-hydroxycyclopentyl]methyl ester (ML792) was bought from Cayman Chemical (Cat. No. 36820). For cell lysis, a protease inhibitor cocktail was purchased from Selleckchem (Cat. No. B14002), while N-Ethylmaleimide (NEM, Cat. No. 04259-5G) was bought from Sigma-Aldrich.

All the antibodies used in this research were tested and validated by the manufacturer. The antibodies used were: Rabbit anti-SUMO2/3 (1:750 WB, 1:200 IF; Sigma, Cat. No. S9571), Rabbit anti-HA (1:500 WB, 1:1000 IF; BioLegend, Cat. No. 902302), Mouse anti-HA (1:1 000 IF; BioLegend, Cat. No. 901503), Mouse anti-myc (1:500 IP; New England Biolabs, Cat. No. 2276S), Rabbit anti-PML (1:200 IF; Thermofisher, Cat. No. A301-167A), Rabbit anti-GFP (1:1000 WB; Invitrogen, Cat. No. A-6455), Rabbit anti-53BP1(1:200, ThermoFisher, Cat. No. A300-272A), HRP-conjugated Donkey anti-rabbit (1:10 000; Cell Signaling, Cat. No. 7074S), AlexaFluor 647-conjugated Goat anti-Rabbit (1:1000, Thermo Fisher Scientific, Cat. No. A-21245), AlexaFluor 488-conjugated Goat anti-Mouse (1:1000, Thermo Fisher Scientific, Cat. No. A-11017, RRID:AB_253408), AlexaFluor 488-conjugated Goat anti-Rabbit (1:1000, Thermo Fisher Scientific, Cat. No. A-11070, RRID:AB_2534114).

#### Constructions

Constructions were generated as described previously.^21^ Briefly, SUMO1^1−97^, SUMO2^1−93,^ and SUMO2 K0 were cloned by Gibson assembly into the BamHI site of a modified pRSFDuet plasmid. SUMO2 Q88R and SUMO2 K0 Q88R were obtained by site-directed mutagenesis. The pET28b/ULP1^403−621^, pET11c/SAE1 and pET28b/SAE2^1−550^ vectors, herein called E1, were obtained from Christopher D. Lima. Ubc9, herein called E2, was synthesized (Integrated DNA Technologies), then cloned into NdeI-XhoI site of pRSFDuet. ZNF (ZNF451^24^^,^^25^^,^^26^^,^^27^^,^^28^^,^^29^^,^^30^^,^^31^^,^^32^^,^^33^^,^^34^^,^^35^^,^^36^^,^^37^^,^^38^^,^^39^^,^^40^^,^^41^^,^^42^^,^^43^^,^^44^^,^^45^^,^^46^^,^^47^^,^^48^^,^^49^^,^^50^^,^^51^^,^^52^^,^^53^^,^^54^^,^^55^) was synthesized (Integrated DNA Technologies). pcDNA3_NSlmb-vhhGFP4 (Addgene plasmid # 35579; http://n2t.net/addgene:35579; RRID:Addgene_35579)^64^ and pcDNA3/p53 WT (Addgene plasmid # 69003; http://n2t.net/addgene:69003; RRID:Addgene_69003)^65^ were gifts from Markus Affolter and David Meek, respectively, and served as sources for the p53 and vhhGFP4 sequences. Vectors pcDNA3.1/p53-GFP-myc-P2A-vhhGFP4-ZNF-HA, and pcDNA3.1/p53-GFP-myc-P2A-vhhGFP4-HA were prepared by inserting the cassettes p53-GFP-myc-P2A-vhhGFP4-ZNF-HA (Figure S3), and p53-GFP-myc-P2A-vhhGFP4-HA (Figure S4) into the HindIII-XhoI site of pcDNA3.1 (+) (Invitrogen). HA-p53, HA-ZNF-p53, VHH-ZNF and p53-GFP, were cloned by Gibson assembly into the BamHI site of a pTRX vector for protein expression in bacteria. HA-p53, V5-p53, and HA-ZNF-p53 were cloned by Gibson assembly into the HindIII-XhoI site of pcDNA3.1 (+) (Invitrogen) for expression in HEK293 cells. Sanger sequencing was used to confirm constructs and mutations.

#### Protein expression in *E.coli* and purification

Proteins were expressed as previously described.^21^ Briefly, plasmids were transformed into competent BL21 DE3 Gold Codon Plus RIL by heat shock. Bacteria were cultured in SB Broth and induced using IPTG overnight at 18°C. Bacteria were then pelleted, resuspended in a sucrose solution, and then flash-frozen in liquid nitrogen. Cell pellets were thawed, sonicated, and then centrifuged at high speed, keeping the soluble fraction for purification.

SAE1/SAE2^1−550^, SUMO1, SUMO2 (and Q88R mutant), SUMO2 K0 (and Q88R mutant), ULP1^403−621^, HA-p53, HA-ZNF-p53, p53-GFP, Ubc9, and ZNF, were purified by gravity flow purifications as previously described.^21^ p53-GFP, and VHH-ZNF, were purified following the same protocol.^21^

The His_6_-tag from SAE1/SAE2^1−550^, SUMO1, SUMO2, and SUMO2 K0, and His_6_-thioredoxin tag from SAE1/SAE2^1−550^, HA-p53, HA-ZNF-p53, p53-GFP, nanobody-ZNF were cleaved overnight at 4°C using 1 μM TEV protease. All proteins were then purified by size exclusion chromatography, as previously described.^21^ Validation of protein purity was done using SDS-Page. Proteins were concentrated by ultrafiltration, and their concentration was determined by absorbance at 280 nm. Proteins were flash-frozen in liquid nitrogen and stored at −80°C until needed.

#### *In vitro* SUMOylation assays

SUMOylation reactions were performed as previously described.^21^ Briefly, 100 nM E1, 100 nM E2, and 50 μM SUMO were used in every reaction. Control reactions also contained 1 μM of p53-GFP. 1 μM of ZNF or VHH-ZNF was added if needed. Alternatively, 1 μM of HA-ZNF-p53 was used instead of both p53-GFP and ZNF. Reactions were initiated by adding ATP and were stopped at each time point by the addition of Laemmli buffer. Samples were loaded on a 12% SDS-Page gel. Migrations were performed at 180V for 55 min, followed by GFP visualization using blue epi illumination. Gels were then stained by Coomassie Blue staining and images were acquired using a ChemiDoc MP (Bio-Rad). Quantifications were performed using ImageLab.

Single-use protein aliquots were used for each assay, which were done in technical triplicates.

#### Transfection of HEK293 cells

For immunoprecipitation assays, cells were transfected in 6-well plates. Specifically, 3 μg of PEI diluted in 100 μL PBS was added to a total of 1 μg DNA plasmid diluted in 100 μL PBS. The mixture was incubated for 20 min at room temperature before cells (1 × 10^6^) were diluted in 2 mL of culture medium and added to the mixture. For SUMO E1 inhibition, cells were treated ML792 (5 μM) or with vehicle (DMSO 0.01%) for 4 h. For immunofluorescence assays, transfection was performed in 24-well plates containing a 12 mm round glass coverslip #1.5 (UltiDent Scientific Inc., St-Laurent, QC, Canada, Cat. No. 170-C12MM). All the volumes and quantities were scaled down by a factor of 5 from those used for 6-well plates to keep the ratio constant between assays.

#### Immunoprecipitation assays

Cell lysis was conducted as previously described,^21^ using 1 mL RIPA buffer (20 mM Tris-HCl pH 7.5, 150 NaCl, 1% Triton X-100, 5 mM EDTA, 0.1% SDS, 1x protease inhibitor cocktail) containing N-Ethylmaleimide (20 mM) per condition. For immunoprecipitation, 1:500 of Myc antibody, and 10 μL of Protein A/G Plus-Agarose resin (Santa Cruz, Cat. No. sc-2003) were added to the supernatants for overnight incubation rotating mixer at 4°C. Washes and elutions were done as previously described^21^

#### Western blotting

All samples were loaded on 12% or 7.5% SDS-polyacrylamide 1 mm gels, except for the anti-SUMO2/3 immunoblot of the immunoprecipitation, which was loaded on a 1.5 mm gel, containing 0.5% 2,2,2-trichloroethanol (TCE) and migrated at 180V for 55 min. Gels were visualized with the stain-free gel protocol of ImageLab, by activating the gel for 45 s, using a ChemiDoc MP. Proteins were then transferred to a 0.45 μm PVDF membrane (Millipore) using Trans-Blot Turbo system (Bio-Rad, Mississauga, ON, Canada) for 7 min, at 25V and 2.5A. For 1.5 mm gels, the transfer time was 10 min instead. Blots were imaged with a ChemiDoc MP using the Stain-free blot protocol of ImageLab. The blocking, primary, and secondary antibody steps were conducted as previously described.^21^

#### Mass spectrometry experiments

##### *In vitro* sample preparation

Reactions were performed as described above in 50 mM Tris-HCl (pH 7.5), 5 mM MgCl_2_, and 3 mM DTT. Reactions were started with 5 mM ATP, and, after 10 min, stopped by incubating at 95°C for 5 min, followed by a snap-freeze in liquid nitrogen. Samples were then prepared as previously described,^11^^,^^21^ by adding ammonium bicarbonate (ABC) to a final concentration of 100 mM. Samples were digested overnight at 37 °C with trypsin, then desalted using C18 StageTips (Cell Signaling Technology, Cat. No. 45943S) according to the manufacturer’s instructions. Samples were dried in a SpeedVac.

##### *In cellula* sample preparation

Detection of SUMOylated lysine residues in HEK293 cells stably expressing 6xHis-SUMO3-Q87R/Q88N was performed as previously described.^11^^,^^21^ Briefly, 48-h transfected cells with pcDNA3/p53-GFP-myc-P2A-vhhGFP4-ZNF-HA, or p53-GFP-myc-P2A-vhhGFP4-HA were lysed in 50 mM Tris-HCl (pH 7.6), 1.5 mM MgCl_2_, 420 mM NaCl, 0.2 mM EDTA, and 25% glycerol, supplemented with 20 mM NEM, 1× protease inhibitor cocktail, and 1× phosphatase inhibitor cocktail (Sigma Aldrich, Cat. No. P0044). Bradford assay was used to determine protein concentration. Immunoprecipitation of myc-tag was performed by using 4 mg of total protein. Beads were washed three times using lysis buffer, then 5 μg trypsin in 2 mL of 50 mM ABC were added to digest the proteins at 37 °C overnight. Samples were dried in a SpeedVac.

#### LC-MS/MS analysis

*In vitro* and *in cellula* SUMO samples were prepared and analyzed as previously described.^11^^,^^21^ Briefly, dried peptides were resuspended in 12 μL of LC buffer A (4% formic acid in water), and 10 μL of the reconstituted samples was injected for analysis. Peptide separation was achieved using a Vanquish *Neo* HPLC system (Thermo Fisher Scientific) equipped with an IonOpticks Aurora Ultimate C18 column (25 cm × 75 μm, 1.7 μm particle size). The peptides were eluted with a linear gradient ranging from 5% to 35% of LC buffer B (0.1% formic acid in acetonitrile) over 51 min at a flow rate of 0.3 μL/min. The eluate was directly analyzed on an Orbitrap Exploris 480 mass spectrometer (Thermo Fisher Scientific) operated in positive ion mode. Full MS scans were acquired at a resolution of 120,000 (m/z 200) over an m/z range of 400–1800, with automatic gain control (AGC) and injection time set to standard parameters. Data-dependent acquisition was carried out in 3-s cycles targeting the most abundant precursor ions, isolated with a 1 Da window. Fragmentation was performed using higher-energy collisional dissociation (HCD) at a normalized collision energy of 30%, and MS/MS spectra were collected at 60,000 resolution (m/z 200) with a normalized AGC target of 100% and a maximum injection time of 123 ms.

#### Data processing

Raw MS/MS spectra were analyzed using MaxQuant software (version 2.2.0) against the UniProt/Swiss-Prot human protein database (release dated November 06, 2020) with additional protein sequences added below.

##### >sp|MycTP53GFP

MEEPQSDPSVEPPLSQETFSDLWKLLPENNVLSPLPSQAMDDLMLSPDDIEQWFTEDPGPDEAPRMPEAAPPVAPAPAAPTPAAPAPAPSWPLSSSVPSQKTYQGSYGFRLGFLHSGTAKSVTCTYSPALNKMFCQLAKTCPVQLWVDSTPPPGTRVRAMAIYKQSQHMTEVVRRCPHHERCSDSDGLAPPQHLIRVEGNLRVEYLDDRNTFRHSVVVPYEPPEVGSDCTTIHYNYMCNSSCMGGMNRRPILTIITLEDSSGNLLGRNSFEVRVCACPGRDRRTEEENLRKKGEPHHELPPGSTKRALPNNTSSSPQPKKKPLDGEYFTLQIRGRERFEMFRELNEALELKDAQAGKEPGGSRAHSSHLKSKKGQSTSRHKKLMFKTEGPDSDEFGGSGGGAAAVSKGEELFTGVVPILVELDGDVNGHKFSVSGEGEGDATYGKLTLKFICTTGKLPVPWPTLVTTLTYGVQCFSRYPDHMKQHDFFKSAMPEGYVQERTIFFKDDGNYKTRAEVKFEGDTLVNRIELKGIDFKEDGNILGHKLEYNYNSHNVYIMADKQKNGIKVNFKIRHNIEDGSVQLADHYQQNTPIGDDPVLLPDNHYLSTQSALSKDPNEKRDHMVLLEFVTAAGITLGGGSGSEQKLISEEDL.

##### >sp|MycTP53ZNFGFP

MEEPQSDPSVEPPLSQETFSDLWKLLPENNVLSPLPSQAMDDLMLSPDDIEQWFTEDPGPDEAPRMPEAAPPVAPAPAAPTPAAPAPAPSWPLSSSVPSQKTYQGSYGFRLGFLHSGTAKSVTCTYSPALNKMFCQLAKTCPVQLWVDSTPPPGTRVRAMAIYKQSQHMTEVVRRCPHHERCSDSDGLAPPQHLIRVEGNLRVEYLDDRNTFRHSVVVPYEPPEVGSDCTTIHYNYMCNSSCMGGMNRRPILTIITLEDSSGNLLGRNSFEVRVCACPGRDRRTEEENLRKKGEPHHELPPGSTKRALPNNTSSSPQPKKKPLDGEYFTLQIRGRERFEMFRELNEALELKDAQAGKEPGGSRAHSSHLKSKKGQSTSRHKKLMFKTEGPDSDEFGGSGGGAAAVSKGEELFTGVVPILVELDGDVNGHKFSVSGEGEGDATYGKLTLKFICTTGKLPVPWPTLVTTLTYGVQCFSRYPDHMKQHDFFKSAMPEGYVQERTIFFKDDGNYKTRAEVKFEGDTLVNRIELKGIDFKEDGNILGHKLEYNYNSHNVYIMADKQKNGIKVNFKIRHNIEDGSVQLADHYQQNTPIGDDPVLLPDNHYLSTQSALSKDPNEKRDHMVLLEFVTAAGITLGGGSGSEQKLISEEDLAAAGSGATNFSLLKQAGDVEENPGPGSMDQVQLVESGGALVQPGGSLRLSCAASGFPVNRYSMRWYRQAPGKEREWVAGMSSAGDRSSYEDSVKGRFTISRDDARNTVYLQMNSLKPEDTAVYYCNVNVGFEYWGQGTQVTVSSGGSGGGGSDENEDDIQFVSEGPLRPVLEYIDLVSSDDEEGTYPYDVPDYA.

##### >sp|HisTP53

MGSSHHHHHHSSGTSDKIIHLTDDSFDTDVLKADGAILVDFWAEWCGPCKMIAPILDEIADEYQGKLTVAKLNIDQNPGTAPKYGIRGIPTLLLFKNGEVAATKVGALSKGQLKEFLDANLAGTENLYFQGSMEEPQSDPSVEPPLSQETFSDLWKLLPENNVLSPLPSQAMDDLMLSPDDIEQWFTEDPGPDEAPRMPEAAPPVAPAPAAPTPAAPAPAPSWPLSSSVPSQKTYQGSYGFRLGFLHSGTAKSVTCTYSPALNKMFCQLAKTCPVQLWVDSTPPPGTRVRAMAIYKQSQHMTEVVRRCPHHERCSDSDGLAPPQHLIRVEGNLRVEYLDDRNTFRHSVVVPYEPPEVGSDCTTIHYNYMCNSSCMGGMNRRPILTIITLEDSSGNLLGRNSFEVRVCACPGRDRRTEEENLRKKGEPHHELPPGSTKRALPNNTSSSPQPKKKPLDGEYFTLQIRGRERFEMFRELNEALELKDAQAGKEPGGSRAHSSHLKSKKGQSTSRHKKLMFKTEGPDSDGGSGGGGSMASKGEELFTGVVPILVELDGDVNGHKFSVSGEGEGDATYGKLTLKFICTTGKLPVPWPTLVTTFSYGVQCFSRYPDHMKRHDFFKSAMPEGYVQERTISFKDDGNYKTRAEVKFEGDTLVNRIELKGIDFKEDGNILGHKLEYNYNSHNVYITADKQKNGIKANFKIRHNIEDGSVQLADHYQQNTPIGDGPVLLPDNHYLSTQSALSKDPNEKRDHMVLLEFVTAAGITHGMDELYK.

The precursor mass tolerance was set to 20 ppm for the initial search and 4.5 ppm for the main search, while fragment ion tolerance was defined at 7.5 ppm. Trypsin/P was selected as the proteolytic enzyme, allowing up to three missed cleavages. Carbamidomethylation of cysteine residues was defined as a fixed modification. Variable modifications included methionine oxidation, N-terminal acetylation, and the SUMO2 remnant (+QQTGG) for *in vitro* SUMO samples; and methionine oxidation, asparagine/glutamine deamidation, serine/threonine/tyrosine phosphorylation, N-terminal acetylation, and the SUMO3 remnant (+NQTGG) for *in cellula* SUMO assays. Peptides with a minimum length of six amino acids were considered, and a false discovery rate (FDR) of 1% was applied at both the peptide and protein levels. Only modification sites with localization probabilities greater than 0.75 were included in subsequent analyses.

#### Immunostaining

Transfected cells were washed using phosphate-buffered saline (PBS) and fixed using 4% paraformaldehyde (PFA)/4% sucrose in PBS for 15 min 0.25% Triton X-100 in PBS was added for 15 min to permeabilize cells. Non-target reactive sites were blocked using 10% normal goat serum (NGS) in PBS for 1 h. Coverslips were then incubated with the diluted primary antibody in 3% NGS/PBS for 1 h. Coverslips were washed three times with PBS, then incubated with secondary antibodies diluted in 3% NGS/PBS for 1 h. Coverslips were again washed three times with PBS, mounted using ProLong Diamond Antifade (Thermo Fisher Scientific, Cat. No. P36961), and kept at 4 °C once dry until needed.

#### Imaging

Images were acquired using an inverted epi-fluorescence microscope Olympus IX83 equipped with a U Plan S-Apo 60×/1.35 numerical aperture oil objective (Olympus Canada Inc., Richmond Hill, ON, Canada), the X-Cite Xylis 365 LED-based illumination source (Excelitas Technologies Corp., Waltham, MA, USA), and the Zyla 4.2 Plus sCMOS camera (Andor, Concord, MA, USA). The filters and dichroic mirror have been thoroughly described in Cabana et al.^68^ z stack images were taken at 0.28 μm for 7–12 optical slices. The resolution was set at 2048 × 2048 pixels. Images were deconvoluted using the Olympus 3D Deconvolution feature in the Olympus CellSens Dimension software.

### Quantification and statistical analysis

All statistical analyses and graphics were generated using Prism 8.0.1.

For *in vitro* analyses from Figures 1, 2, and 4, quantifications were performed using ImageLab. The relative abundance of SUMOylated and non SUMOylated substrate was determined by measuring the band intensity of unmodified substrate (No SUMO), monoSUMOylation, diSUMOylation, triSUMOylation, and poly/multiSUMOylation (polySUMO). DiSUMO2 was quantified by measuring the band intensity corresponding to diSUMO2. All results are mean ± SD from *N* = 3.

For immunoprecipitation assays quantifications from Figure 5, quantifications were performed using ImageLab. the percentage of SUMOylated protein was estimated by dividing the band intensity of the monoSUMOylation band by the total signal of unmodified and monoSUMOylated p53-GFP from the anti-GFP immunoblot of the immunoprecipitation. Statistical analysis was performed using a multiple comparison two-way ANOVA followed by a Tukey test. All results are mean ± SD from *N* = 3. Statistical details can be found in the legend of Figure 5.

To analyze the number of foci of each individual transfected cell of microscopy images of Figures 6, 7, And 8, the channels were first split, and the optical sections were Z-stacked using maximum intensity projection in Fiji. Multiple thresholds were tested to minimize background signal while preserving foci signals using a single condition for each marker and each independent experiment. Appropriate brightness and contrast were then standardized across all conditions for each marker and independent experiment using the same minimum and maximum values in the “Set Display Range” option of Fiji’s Brightness/Contrast adjustment. Fiji’s “Max Entropy” threshold was then applied, and the images were converted to a mask. The Watershed function was used to separate closely adjacent foci. To avoid including artifacts that could have been introduced by deconvolution because of the low signal-to-noise ratios of some conditions,^69^ only foci greater than 0.2 μm for SUMO2/3, GFP, and 53BP1 and 0.25 μm for PML were counted using the Fiji’s Analyze particles and Measure functions. Composite images with ROIs were generated, saved and used to analyze the percentage of SUMO, PML, or 53BP1 foci overlapping GFP foci. Specifically, the two composite channels of an image were multiplied by one another using Fiji’s Image Calculator function to create a new image. If one composite contained no signal, the other composite signal was multiplied by zero, removing non-overlapping signals. Foci in the new image were compared with the ROIs from the composite images to confirm and quantify overlap. The percentage of overlapping foci was calculated by dividing the number of overlapping foci by the total number of foci. When multiple groups with two grouping variables were compared, statistical difference was determined using repeated measures two-way ANOVA with uncorrected Fisher’s LSD multiple comparisons test to compare means with others in its row and its column. All results are mean ± SEM from *N* = 3. Statistical methods can be found in the legends of Figures 6, 7, and 8.
