# Supplementary material for: A ZNF-nanobody fusion reveals SUMOylation-dependent changes in p53 protein localization
Source: iScience. 2026 Apr 9;29(5):115673. doi: 10.1016/j.isci.2026.115673 (PMC13127394; doi:10.1016/j.isci.2026.115673)
Supplement: Document S1. Figures S1–S4 [file mmc1.pdf]

iScience, Volume *29*

Supplemental information

A ZNF-nanobody fusion reveals SUMOylation-dependent changes in p53 protein localization

Antoine Y. Bouchard, Valérie C. Cabana, Julien Plamondon, Chongyang Li (李重阳), Anaïs J.I. Vivet, Sylvie Mader, Pierre Thibault, Marc P. Lussier, and Laurent Cappadocia

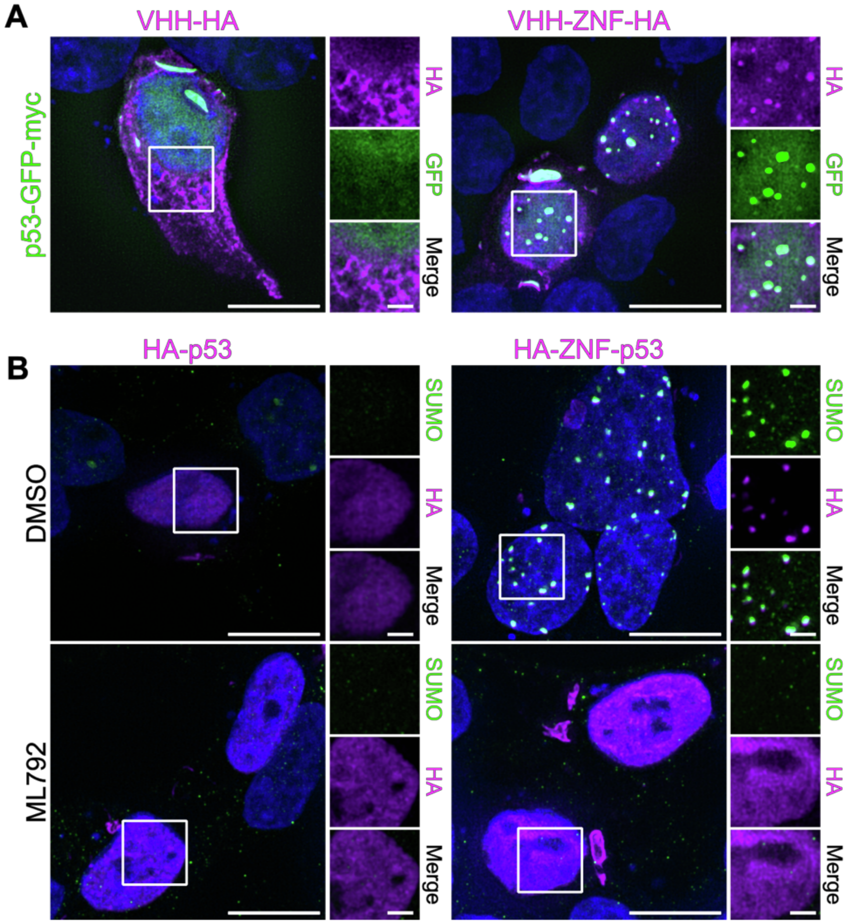

Supplementary Figure 1: VHH-HA colocalize with p53-GFP-myc and induce the same localization changes as our recently developed small ZNF tag, related to Figure 6.

Representative (N=3) immunofluorescence images show HEK293 cells expressing (A) p53-GFP-Myc-P2A-VHH-HA or p53-GFP-Myc-P2A-VHH-ZNF-HA, (B) HA-p53 or HA-ZNF-p53. (B) 48 hours post-transfection, cells were treated with 5 µM ML792 or vehicle (0,01% DMSO) for 4 hours. (A-B) Cells were fixed, permeabilized, and stained using anti-HA primary antibody to detect (A) VHH or (B) p53 and (B) anti-SUMO2/3 primary antibody against endogenous protein. Scale bars indicate 10 µm for whole cell image and 2 µm for higher magnification.

To generate pcDNA3.1/p53-GFP-myc-P2A-VHH-ZNF-HA and pcDNA3.1/p53-GFP-myc-P2A-VHH-HA	
Cassette_HindIII_F	CACTATAGGGAGACCCAAGCTTGAATTCGGTGGTTCTGGCGGTGGAGCGGCCGCAGGATCTGGAGCAAC
Cassette_XhoI_R	TCTAGATGCATGCTCGAGGGATCCAGGCCCGGGATTCTCC
GFP_NotI_F	GTGGTTCTGGCGGTGGAGCGGCCGCAGTGAGCAAGGGCGAGG
GFP_NotI_R	TGTTGCTCCAGATCCTGCGGCCGCGGAGCCGGAGCCCCC
GFP_myc_NotI_R	TGTTGCTCCAGATCCTGCGGCCGCCAGATCCTCTTCTGAGATGAGTTTTTGTTCGGAGCCGGAGCCCCC
VHH-ZNF_BamHI_F	GAGAATCCCGGGCCT GGATCC ATGGATCAAGTCCAACTGGTG
VHH-ZNF-HA_BamHI_R	CTAGATGCATGCTCGAGGGATCATCAAGCGTAATCTGGAACATCGTATGGGTAGGTACCCTCTTCATCATCACTGCTGACC
VHH_HA_BamHI_R	GTCGTCTTCATTTTCATCGGATCCTCAAGCGTAATCTGGAACATCGTATGGGTAGGTACCGCTGGAGACGGTGACCTGGG
p53_EcoRI_F	GGGAGACCCAAGCTTGAATTCGCCACCATGGAGGAGCCGCAGTC
p53_EcoRI_R	CCGCCAGAACCACCGAATTCGTCTGAGTCAGGCCC
**To generate pTRX/VHH-ZNF-HA**	
pTrx-vhhGFP4_F,	GGTACCGAAAACTTGTATTTCCAGGGATCCATGGATCAAGTCCAACTGGTGGAG
pTrx-ZNF_R,	GCTTGTCGACGGAGCTCGAATTCGGATCCTCACTCTTCATCATCACTGC
**To generate pTRX/p53-GFP**	
BamHI-p53F	GAAAACTTGTATTTCCAGGGATCCATGGAGGAGCCGCAGTCAGATCC
pTrx-GFP_R	GCTTGTCGACGGAGCTCGAATTCGGATCCTCATTTGTAGAGCTCATCCATGCC

Supplementary Figure 2: List of oligonucleotides for cloning, related to the STAR Method. The oligos were ordered from Integrated DNA Technologies (IDT) and used for the various cloning.

Cassette p53-GFP-myc-P2A-vhhGFP4-ZNF-HA

*AAGCTT*GAATTCGCCACCATGGAGGAGCCGCAGTCAGATCCTAGCGTCGAGCCCCCTCTGAGTCAGGAAACATTTTCAGACCTATGGAA
ACTACTTCCTGAAAACAACGTTCTGTCCCCCTTGCCGTCCCAAGCAATGGATGATTTGATGCTGTCCCCGGACGATATTGAACAATGGT
TCACTGAAGACCCAGGTCCAGATGAAGCTCCCAGAATGCCAGAGGCTGCTCCCCCCGTGGCCCCTGCACCAGCAGCTCCTACACCGGCG
GCCCCTGCACCAGCCCCCTCCTGGCCCCTGTCATCTTCTGTCCCTTCCCAGAAAACCTACCAGGGCAGCTACGGTTTCCGTCTGGGCTT
CTTGCATTCTGGGACAGCCAAGTCTGTGACTTGCACGTACTCCCCTGCCCTCAACAAGATGTTTTGCCAACTGGCCAAGACCTGCCCTG
TGCAGCTGTGGGTTGATTCCACACCCCCGCCCGGCACCCGCGTCCGCGCCATGGCCATCTACAAGCAGTCACAGCACATGACGGAGGTT
GTGAGGCGCTGCCCCCACCATGAGCGCTGCTCAGATAGCGATGGTCTGGCCCCTCCTCAGCATCTTATCCGAGTGGAAGGAAATTTGCG
TGTGGAGTATTTGGATGACAGAAACACTTTTCGACATAGTGTGGTGGTGCCCTATGAGCCGCCTGAGGTTGGCTCTGACTGTACCACCA
TCCACTACAACTACATGTGTAACAGTTCCTGCATGGGCGGCATGAACCGGAGGCCCATCCTCACCATCATCACACTGGAAGACTCCAGT
GGTAATCTACTGGGACGGAACAGCTTTGAGGTGCGTGTTTGTGCCTGTCCTGGGAGAGACCGGCGCACAGAGGAAGAGAATCTCCGCAA
GAAAGGGGAGCCTCACCACGAGCTGCCCCCAGGGAGCACTAAGCGAGCACTGCCCAACAACACCAGCTCCTCTCCCCAGCCAAAGAAGA
AACCACTGGATGGAGAATATTTCACCCTTCAGATCCGTGGGCGTGAGCGCTTCGAGATGTTCCGAGAGCTGAATGAGGCCTTGGAACTC
AAGGATGCCCAGGCTGGGAAGGAGCCAGGGGGGAGCAGGGCTCACTCCAGCCACCTGAAGTCCAAAAAGGGTCAGTCTACCTCCCGCCA
TAAAAAACTCATGTTCAAGACAGAAGGGCCTGACTCAGACGAATTCGGTGGTTCTGGCGGTGGAGCGGCCGCAGTGAGCAAGGGCGAGG
AGCTGTTCACCGGGGTGGTGCCCATCCTGGTCGAGCTGGACGGCGACGTAAACGGCCACAAGTTCAGCGTGTCCGGCGAGGGCGAGGGC
GATGCCACCTACGGCAAGCTGACCCTGAAGTTCATCTGCACCACCGGCAAGCTGCCCGTGCCCTGGCCCACCCTCGTGACCACCCTGAC
CTACGGCGTGCAGTGCTTCAGCCGCTACCCCGACCACATGAAGCAGCACGACTTCTTCAAGTCCGCCATGCCCGAAGGCTACGTCCAGG
AGCGCACCATCTTCTTCAAGGACGACGGCAACTACAAGACCCGCGCCGAGGTGAAGTTCGAGGGCGACACCCTGGTGAACCGCATCGAG
CTGAAGGGCATCGACTTCAAGGAGGACGGCAACATCCTGGGGCACAAGCTGGAGTACAACTACAACAGCCACAACGTCTATATCATGGC
CGACAAGCAGAAGAACGGCATCAAGGTGAACTTCAAGATCCGCCACAACATCGAGGACGGCAGCGTGCAGCTCGCCGACCACTACCAGC
AGAACACCCCCATCGGCGACGGCCCCGTGCTGCTGCCCGACAACCACTACCTGAGCACCCAGTCCGCCCTGAGCAAAGACCCCAACGAG
AAGCGCGATCACATGGTCCTGCTGGAGTTCGTGACCGCCGCCGGGATCACTCTCGGCGGGGGCTCCGGCTCCGAACAAAAACTCATCTC
AGAAGAGGATCTGGCGGCCGCAGGATCTGGAGCAACAAACTTCTCACTACTCAAACAAGCAGGTGACGTGGAGGAGAATCCCGGGCCTG
GATCCATGGATCAAGTCCAACTGGTGGAGTCTGGTGGCGCTTTGGTGCAGCCAGGTGGCTCTCTGCGTTTGTCCTGTGCCGCTTCTGGC
TTCCCAGTGAACCGCTATTCCATGCGCTGGTATCGCCAGGCTCCAGGCAAAGAGCGTGAGTGGGTAGCCGGTATGTCCAGCGCGGGTGA
TCGTAGCTCCTATGAAGACTCCGTGAAGGGCCGTTTCACCATCAGCCGTGACGATGCCCGTAACACGGTGTATCTGCAAATGAACAGCT
TGAAACCTGAAGATACGGCCGTGTATTACTGTAATGTGAACGTGGGCTTCGAGTATTGGGGCCAAGGCACCCAGGTCACCGTCTCCAGC
GGTGGTTCTGGCGGTGGAGGATCCGATGAAAATGAAGACGACATTCAGTTTGTCAGTGAAGGACCATTACGACCTGTTCTTGAATACAT
TGATCTGGTCAGCAGTGATGATGAAGAGGGTACCTACCCATACGATGTTCCAGATTACGCTTGATGATCC*CTCGAG*

Translation of cassette p53-GFP-myc-P2A-vhhGFP4-ZNF-HA

MEEPQSDPSVEPPLSQETFSDLWKLLPENNVLSPLPSQAMDDLMLSPDDIEQWFTEDPGPDEAPRMPEAAPPVAPAPAAPTPAAPAPAP
SWPLSSSVPSQKTYQGSYGFRLGFLHSGTAKSVTCTYSPALNKMFCQLAKTCPVQLWVDSTPPPGTRVRAMAIYKQSQHMTEVVRRCPH
HERCSDSDGLAPPQHLIRVEGNLRVEYLDDRNTFRHSVVVPYEPPEVGSDCTTIHYNYMCNSSCMGGMNRRPILTIITLEDSSGNLLGR
NSFEVRVCACPGRDRRTEEENLRKKGEPHHELPPGSTKRALPNNTSSSPQPKKKPLDGEYFTLQIRGRERFEMFRELNEALELKDAQAG
KEPGGSRAHSSHLKSKKGQSTSRHKKLMFKTEGPDSDEFGGSGGGAAAVSKGEELFTGVVPILVELDGDVNGHKFSVSGEGEGDATYGK
LTLKFICTTGKLPVPWPTLVTTLTYGVQCFSRYPDHMKQHDFFKSAMPEGYVQERTIFFKDDGNYKTRAEVKFEGDTLVNRIELKGIDF
KEDGNILGHKLEYNYNSHNVYIMADKQKNGIKVNFKIRHNIEDGSVQLADHYQQNTPIGDGPVLLPDNHYLSTQSALSKDPNEKRDHMV
LLEFVTAAGITLGGGSGSEQKLISEEDLAAAGSGATNFSLLKQAGDVEENPGPGSMDQVQLVESGGALVQPGGSLRLSCAASGFPVNRY
SMRWYRQAPGKEREWVAGMSSAGDRSSYEDSVKGRFTISRDDARNTVYLQMNSLKPEDTAVYYCNVNVGFEYWGQGTQVTVSSGGSGGG
GSDENEDDIQFVSEGPLRPVLEYIDLVSSDDEEGTYPYDVPDYA*

Supplementary Figure 3: Constructions of the VHH-ZNF-HA plasmid used in cellula, related to the STAR Method. Cassette inserted into the HindIII-XhoI sites of pcDNA3.1 to generate pcDNA3.1/p53-GFP-myc-P2A-vhhGFP4-ZNF-HA. DNA sequences are indicated on top and their translation is presented at the bottom. Colors indicate the position of p53, GFP, myc, P2A, vhhGFP4, ZNF and HA. Italics indicate the position of the HindIII and XhoI restriction sites.

Cassette p53-GFP-myc-P2A-vhhGFP4-HA

*AAGCTT*GAATTCGCCACCATGGAGGAGCCGCAGTCAGATCCTAGCGTCGAGCCCCCTCTGAGTCAGGAAACATTTTCAGACCTATGGAA
ACTACTTCCTGAAAACAACGTTCTGTCCCCCTTGCCGTCCCAAGCAATGGATGATTTGATGCTGTCCCCGGACGATATTGAACAATGGT
TCACTGAAGACCCAGGTCCAGATGAAGCTCCCAGAATGCCAGAGGCTGCTCCCCCCGTGGCCCCTGCACCAGCAGCTCCTACACCGGCG
GCCCCTGCACCAGCCCCCTCCTGGCCCCTGTCATCTTCTGTCCCTTCCCAGAAAACCTACCAGGGCAGCTACGGTTTCCGTCTGGGCTT
CTTGCATTCTGGGACAGCCAAGTCTGTGACTTGCACGTACTCCCCTGCCCTCAACAAGATGTTTTGCCAACTGGCCAAGACCTGCCCTG
TGCAGCTGTGGGTTGATTCCACACCCCCGCCCGGCACCCGCGTCCGCGCCATGGCCATCTACAAGCAGTCACAGCACATGACGGAGGTT
GTGAGGCGCTGCCCCCACCATGAGCGCTGCTCAGATAGCGATGGTCTGGCCCCTCCTCAGCATCTTATCCGAGTGGAAGGAAATTTGCG
TGTGGAGTATTTGGATGACAGAAACACTTTTCGACATAGTGTGGTGGTGCCCTATGAGCCGCCTGAGGTTGGCTCTGACTGTACCACCA
TCCACTACAACTACATGTGTAACAGTTCCTGCATGGGCGGCATGAACCGGAGGCCCATCCTCACCATCATCACACTGGAAGACTCCAGT
GGTAATCTACTGGGACGGAACAGCTTTGAGGTGCGTGTTTGTGCCTGTCCTGGGAGAGACCGGCGCACAGAGGAAGAGAATCTCCGCAA
GAAAGGGGAGCCTCACCACGAGCTGCCCCCAGGGAGCACTAAGCGAGCACTGCCCAACAACACCAGCTCCTCTCCCCAGCCAAAGAAGA
AACCACTGGATGGAGAATATTTCACCCTTCAGATCCGTGGGCGTGAGCGCTTCGAGATGTTCCGAGAGCTGAATGAGGCCTTGGAACTC
AAGGATGCCCAGGCTGGGAAGGAGCCAGGGGGGAGCAGGGCTCACTCCAGCCACCTGAAGTCCAAAAAGGGTCAGTCTACCTCCCGCCA
TAAAAAACTCATGTTCAAGACAGAAGGGCCTGACTCAGACGAATTCGGTGGTTCTGGCGGTGGAGCGGCCGCAGTGAGCAAGGGCGAGG
AGCTGTTCACCGGGGTGGTGCCCATCCTGGTCGAGCTGGACGGCGACGTAAACGGCCACAAGTTCAGCGTGTCCGGCGAGGGCGAGGGC
GATGCCACCTACGGCAAGCTGACCCTGAAGTTCATCTGCACCACCGGCAAGCTGCCCGTGCCCTGGCCCACCCTCGTGACCACCCTGAC
CTACGGCGTGCAGTGCTTCAGCCGCTACCCCGACCACATGAAGCAGCACGACTTCTTCAAGTCCGCCATGCCCGAAGGCTACGTCCAGG
AGCGCACCATCTTCTTCAAGGACGACGGCAACTACAAGACCCGCGCCGAGGTGAAGTTCGAGGGCGACACCCTGGTGAACCGCATCGAG
CTGAAGGGCATCGACTTCAAGGAGGACGGCAACATCCTGGGGCACAAGCTGGAGTACAACTACAACAGCCACAACGTCTATATCATGGC
CGACAAGCAGAAGAACGGCATCAAGGTGAACTTCAAGATCCGCCACAACATCGAGGACGGCAGCGTGCAGCTCGCCGACCACTACCAGC
AGAACACCCCCATCGGCGACGGCCCCGTGCTGCTGCCCGACAACCACTACCTGAGCACCCAGTCCGCCCTGAGCAAAGACCCCAACGAG
AAGCGCGATCACATGGTCCTGCTGGAGTTCGTGACCGCCGCCGGGATCACTCTCGGCGGGGGCTCCGGCTCCGAACAAAAACTCATCTC
AGAAGAGGATCTGGCGGCCGCAGGATCTGGAGCAACAAACTTCTCACTACTCAAACAAGCAGGTGACGTGGAGGAGAATCCCGGGCCTG
GATCCATGGATCAAGTCCAACTGGTGGAGTCTGGTGGCGCTTTGGTGCAGCCAGGTGGCTCTCTGCGTTTGTCCTGTGCCGCTTCTGGC
TTCCCAGTGAACCGCTATTCCATGCGCTGGTATCGCCAGGCTCCAGGCAAAGAGCGTGAGTGGGTAGCCGGTATGTCCAGCGCGGGTGA
TCGTAGCTCCTATGAAGACTCCGTGAAGGGCCGTTTCACCATCAGCCGTGACGATGCCCGTAACACGGTGTATCTGCAAATGAACAGCT
TGAAACCTGAAGATACGGCCGTGTATTACTGTAATGTGAACGTGGGCTTCGAGTATTGGGGCCAAGGCACCCAGGTCACCGTCTCCAGC
GGTACCTACCCATACGATGTTCCAGATTACGCTTGATGATCC*CTCGAG*

Translation of cassette p53-GFP-myc-P2A-vhhGFP4-HA
MEEPQSDPSVEPPLSQETFSDLWKLLPENNVLSPLPSQAMDDLMLSPDDIEQWFTEDPGPDEAPRMPEAAPPVAPAPAAPTPAAPAPAP
SWPLSSSVPSQKTYQGSYGFRLGFLHSGTAKSVTCTYSPALNKMFCQLAKTCPVQLWVDSTPPPGTRVRAMAIYKQSQHMTEVVRRCPH
HERCSDSDGLAPPQHLIRVEGNLRVEYLDDRNTFRHSVVVPYEPPEVGSDCTTIHYNYMCNSSCMGGMNRRPILTIITLEDSSGNLLGR
NSFEVRVCACPGRDRRTEEENLRKKGEPHHELPPGSTKRALPNNTSSSPQPKKKPLDGEYFTLQIRGRERFEMFRELNEALELKDAQAG
KEPGGSRAHSSHLKSKKGQSTSRHKKLMFKTEGPDSDEFGGSGGGAAAVSKGEELFTGVVPILVELDGDVNGHKFSVSGEGEGDATYGK
LTLKFICTTGKLPVPWPTLVTTLTYGVQCFSRYPDHMKQHDFFKSAMPEGYVQERTIFFKDDGNYKTRAEVKFEGDTLVNRIELKGIDF
KEDGNILGHKLEYNYNSHNVYIMADKQKNGIKVNFKIRHNIEDGSVQLADHYQQNTPIGDGPVLLPDNHYLSTQSALSKDPNEKRDHMV
LLEFVTAAGITLGGGSGSEQKLISEEDLAAAGSGATNFSLLKQAGDVEENPGPGSMDQVQLVESGGALVQPGGSLRLSCAASGFPVNRY
SMRWYRQAPGKEREWVAGMSSAGDRSSYEDSVKGRFTISRDDARNTVYLQMNSLKPEDTAVYYCNVNVGFEYWGQGTQVTVSSGTYPYD
VPDYA

Supplementary Figure 4: Constructions of the VHH-HA plasmid used in cellula, related to the STAR Method. Cassette inserted into the HindIII-XhoI sites of pcDNA3.1 to generate pcDNA3.1/p53-GFP-myc-P2A-vhhGFP4-HA. DNA sequences are indicated on top and their translation is presented at the bottom. Colors indicate the position of p53, GFP, myc, P2A, vhhGFP4, and HA. Italics indicate the position of the HindIII and XhoI restriction sites.
